# Supplementary material for: Rapid traversal of vast chemical space using machine learning-guided docking screens
Source: Nat Comput Sci. 2025 Mar 13;5(4):301–12. doi: 10.1038/s43588-025-00777-x (PMC12021657; doi:10.1038/s43588-025-00777-x)
Supplement: Supplementary file 1 — Supplementary Sections 1 and 2, Tables 1–14, Figs. 1–30, methods and References. [file 43588_2025_777_MOESM1_ESM.pdf]

nature computational science

Supplementary information

https://doi.org/10.1038/s43588-025-00777-x

Rapid traversal of vast chemical space using machine learning-guided docking screens

In the format provided by the authors and unedited

Table of Contents

Supplementary Sections

1. Conformal prediction and molecular docking **S2**
Supp. Figure 1. Overview of the conformal prediction workflow. S3
Supp. Table 1. Protein preparation for molecular docking. S4
Supp. Figure 2. Partial charge redistribution scheme in amino acid residues. S5

2. Hyperparameters, architectures and molecular descriptors **S6**
Supp. Table 2. Model hyperparameters. S7
Supp. Figure 3. Learning rate and weight decay analysis for DNNs. S8
Supp. Figure 4. Architecture analysis for DNNs. S9
Supp. Figure 5. Learning rate analysis for RoBERTa. S10
Supp. Table 3. Sensitivity and training set size - Morgan2. S11
Supp. Table 4. Precision and training set size - Morgan2. S11
Supp. Table 5. Sensitivity and training size - CDDD. S12
Supp. Table 6. Precision and training size - CDDD. S12
Supp. Table 7. Sensitivity and training size - RoBERTa. S13
Supp. Table 8. Precision and training size - RoBERTa. S13
Supp. Table 9. Training and prediction times. S13
Supp. Figure 6. Performance and number of aggregated models. S14
Supp. Figure 7. Performance on imbalanced datasets. S15
Supp. Figure 8. Overview of noise addition. S16
Supp. Figure 9. Performance on noisy datasets. S17
Supp. Figure 10. Performance on non-sensical datasets - labels. S18
Supp. Figure 11. Performance on non-sensical datasets - features. S19
Supp. Figure 12. Non-exchangeable datasets. S20
Supp. Figure 13. Correlation between quality of information and docking rank. S21
Supp. Figure 14. Structural diversity analysis in top-ranked molecules. S22

Supplementary Tables **S23**

Supp. Table 10. Chemical structures and D_2R radioligand displacement data. S23
Supp. Table 11. Chemical novelty of discovered D_2R ligands. S25
Supp. Table 12. Chemical structures and $A_{2A}R$ radioligand displacement data. S26
Supp. Table 13. Chemical novelty of discovered $A_{2A}R$ ligands. S31
Supp. Table 14. Chemical novelty of discovered $A_{2A}R$-D_2R dual target ligand. S30

Supplementary Figures **S31**

Supp. Figure 15. Binding assay curves of discovered D_2R ligands. S31
Supp. Figure 16. Functional assay curves of discovered D_2R ligands. S32
Supp. Figure 17. Ligand enrichment curves for $A_{2A}R$ and D_2R models. S33
Supp. Figure 18. Binding assay curves of discovered $A_{2A}R$ ligands. S34
Supp. Figure 19. LC-MS data for compound 1. S35
Supp. Figure 20. LC-MS data for compound 2. S36
Supp. Figure 21. LC-MS data for compound 3. S37
Supp. Figure 22. LC-MS data for compound 4. S38
Supp. Figure 23. LC-MS data for compound 5. S39
Supp. Figure 24. LC-MS data for compound 6. S40
Supp. Figure 25. ^{1}H-NMR data for compound 1. S41
Supp. Figure 26. ^{1}H-NMR data for compound 2. S42
Supp. Figure 27. ^{1}H-NMR data for compound 3. S43
Supp. Figure 28. ^{1}H-NMR data for compound 4. S44
Supp. Figure 29. ^{1}H-NMR data for compound 5. S45
Supp. Figure 30. ^{1}H-NMR data for compound 6. S46

Supplementary References S47

Supplementary Section 1: Conformal prediction and molecular docking

An overview of the conformal prediction framework is provided in Supplementary Figure 1. The chosen proteins for our benchmarking set represented diverse types of protein folds, binding sites, protein-ligand interactions, and ligand chemotypes. G protein-coupled receptors (GPCRs) were represented by the A_{2A} adenosine receptor ($A_{2A}R$) and the D_2 dopamine receptor (D_2R). The SARS-CoV-2 main protease (M^{pro}), 8-oxoguanine glycosylase 1 (OGG1), ecto-5'-nucleotidase (5'-NT), and AmpC β-lactamase (AmpC) exemplified different types of soluble enzymes. Finally, the Kelch-like ECH-associated protein 1 (KEAP1) and Sortilin (SORT1) represented protein-protein interaction interfaces. Molecular docking performance was optimized for each target by fine-tuning parameters that influence the scoring function in DOCK3.7, enhancing enrichment of known ligands over matched decoys. The selected docking parameters are presented in Supplementary Table 1 and Supplementary Figure 2.

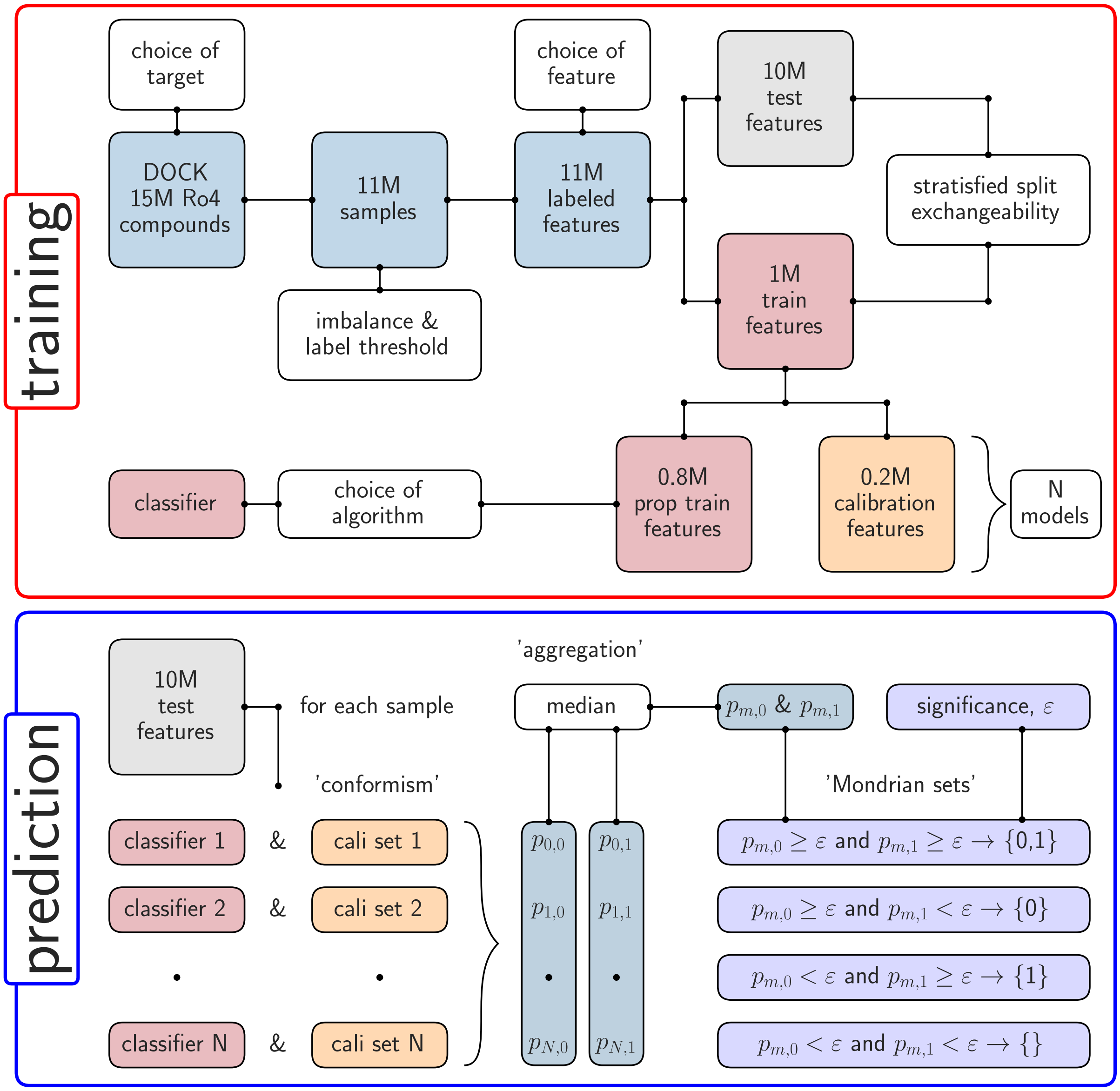

Supplementary Figure 1. Overview of the conformal prediction workflow. After docking to a target of interest, machine learning datasets are obtained through selection of a score threshold, followed by labeling and featurization of samples. Training and test sets are assumed to be exchangeable. The training set is split into a proper training and calibration set, and this process is repeated for each independent model that must be trained. After training the classifiers, each sample in the test set is predicted. The corresponding calibration sets help normalize the outputs given by the classifiers. A pair of p-values (p_1 referring to the confidence the sample belongs to the virtual actives and p_0 referring to the confidence the sample belongs to the virtual inactives class) is obtained after aggregating model outputs by taking median values. After selecting a significance threshold, the sample can be assigned to a set prediction. For binary classifications, Mondrian conformal prediction has four sets that a sample can be categorized into: virtual active {1}, virtual inactive {0}, both = virtual active or inactive {0,1}, and null = no class assignment {}.

Supplementary Table 1. Protein preparation for molecular docking.

Target	Template[a]	Tarted residues[b]	Histidine protonation states	Number of matching spheres	Electrostatic radius[c]	Desolvation radius
$A_{2A}R$	4EIY[1]	N253	δ: 155, 230<br>ε: 75, 250, 306<br>δ+ε: 264, 278	45	1.2 Å	0.3 Å
AmpC	6DPT[2]	S64, Q120<br>N152, A318	ε: 13, 108, 186, 210, 314	45	1.2 Å	0.2 Å
5'-NT	6XUE[3]	N390	δ: 33, 38, 220, 304, 440<br>ε: 103, 243, 375, 383, 437, 456, 518<br>δ+ε: 118	44	1.2 Å	0.4 Å
D_2R	6CM4[4]	None	δ: 393, 398<br>ε: 106	45	1.2 Å	0.25 Å
KEAP1	5FNU[5]	S363, Q530,<br>S555, S602	δ: 436<br>ε: 424, 432, 437, 451, 516, 552, 553, 562, 575	45	1.4 Å	0.2 Å
M^{pro}	6W63[6]	H163, G143,<br>E166	δ: 64, 80<br>ε: 41, 163, 164, 172, 246	64	1.2 Å	0.3 Å
OGG1	6G3Y[7]	G42	δ: 10, 13, 54, 97, 112, 179, 185, 195, 270, 276, 282<br>ε: 119, 237	45	Default (1.9 Å)	None
SORT1	6X48[8]	Y318	δ: 68, 98, 360, 458, 490<br>ε: 70, 182, 220, 295, 331, 406, 428, 430, 506, 590, 664	45	1.6 Å	None
$A_{2A}R$	8GNE[9]	N253	δ: 155, 230, 266<br>ε: 75, 250, 306<br>δ+ε: 278	45	1.2 Å	0.3 Å
D_2R	7CMV[10,d]	S197, S193	ε: 106, 393, 398	45	1.2 Å	0.25 Å

[a] PDB accession code. [b] Increase of dipole moments by adding partial charges to atoms, without altering the total charge of the residue. A detailed description of the partial charge redistribution is provided in Supplementary Figure 1. [c] Tangent thin sphere radius. Default refers to low dielectric spheres made by blastermaster's SPHGEN program prior to thin sphere protocols. [d] A detailed description of homology model generation based on the D_3R is given in the methods section.

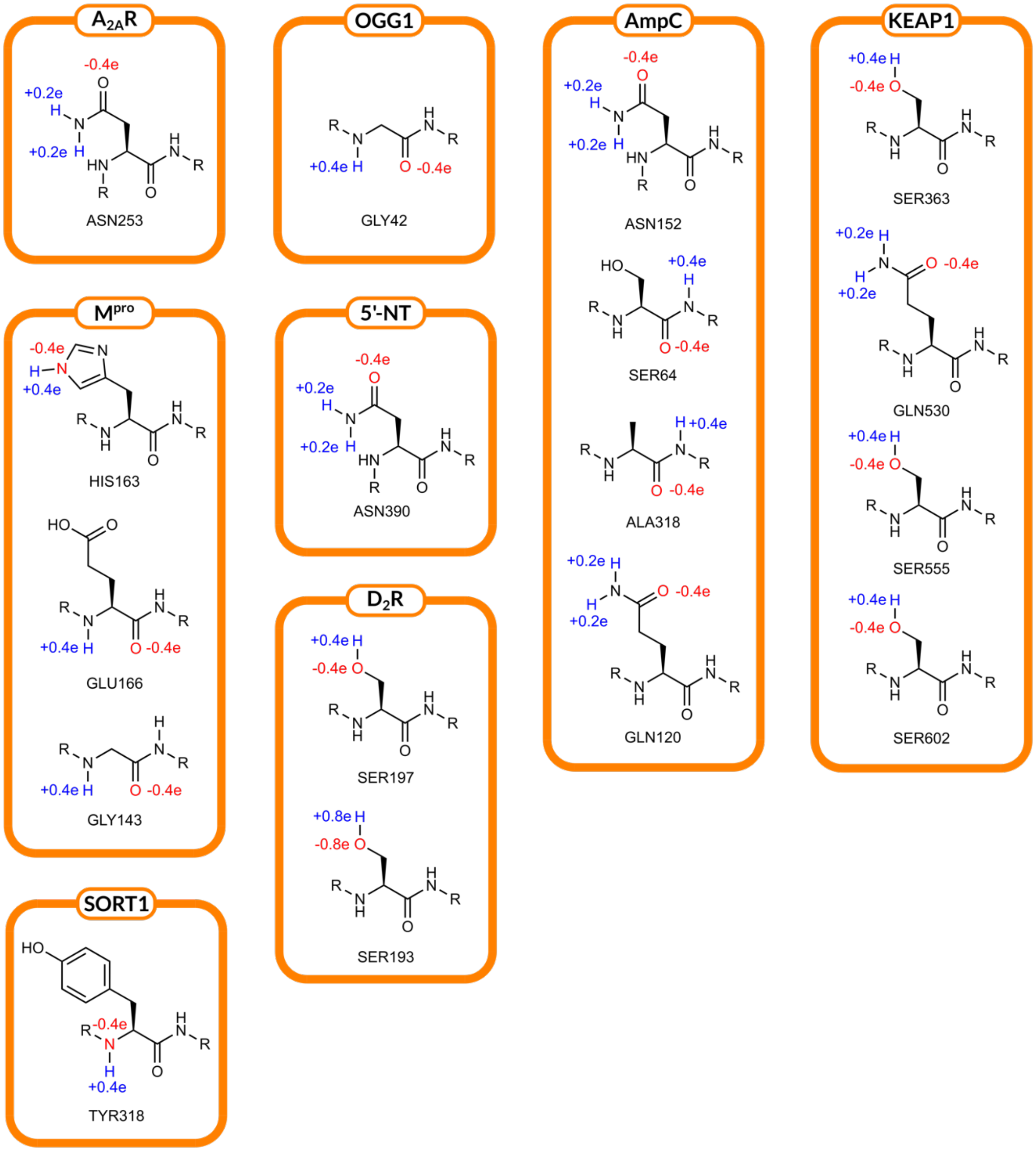

Supplementary Figure 2. Partial charge redistribution in amino acid residues. For each protein target in this study, the increase of dipole moments by adding partial charges to atoms, without altering the total charge of the residue in the preparation of the molecular docking model.

Supplementary Section 2: Evaluation of classifiers and molecular descriptors

CatBoost, DNN and RoBERTa classifiers resulted in consistently high sensitivity values and the three molecular representations showed similar performance. The main differences between the architectures were instead in the precision, significance, and computational cost (Supplementary Tables 3-9). On average, the significance values ranged from 0.15 to 0.18 with prediction efficiencies exceeding 0.99. The CP framework was hence able to classify nearly all evaluated compounds as either virtual active or virtual inactive with an average error rate of 15-18%. Whereas deviations in *validity* (the agreement between the selected significance and resulting error rate) are often observed in applications where insufficient data is available[11], the performance of the CP on molecular docking data resulted in the expected error rate for all targets in the benchmarking set (Figure 2c). Analysis of the results for each protein in the benchmarking set demonstrated that performance was target-dependent, with sensitivity values ranging from 0.76 to 0.96 (Supplementary Tables 3-8). As 100000 compounds in the test set belonged to the actives class, a maximal reduction of 100-fold could be achieved if all compounds were correctly classified.

The largest database reduction was obtained for AmpC, a beta-lactamase targeted for the development of antibiotics.[12] For AmpC, 474646 out of the 10 million compounds in the test set were assigned to the virtual active class, corresponding to a 21-fold database reduction, and 96% of the true virtual actives were among these. The worst performance was obtained for the target M^{pro}, which is a viral protease relevant for development of drugs for treatment of COVID-19.[13] In this case, the database was reduced by four-fold and 76% of the true virtual actives were identified. The target dependent results of machine learning accelerated protocols have been observed previously, and analysis of our docking results indicate that the performance is influenced by the nature of the binding site, the diversity of the top-ranked compounds, and the docking score distribution. For example, the top-scoring compounds of open and solvent-exposed binding sites tend to be more structurally diverse, which affects the ability of the classifier to recognize patterns in the docking data.

Increasing the number of classification models from five to ten did not substantially increase the performance of the conformal predictor, and the results were also robust if the size of the minority class (virtual actives) was decreased from 1% to 0.1% (Supplementary Figures 6-7). The introduction of noise in the docking scores did not substantially alter the performance of the predictor (Supplementary Figures 8-9), but scrambling of class labels or features led to complete loss of predictive power (Supplementary Figures 10-11). Exchangeability is a fundamental concept in conformal prediction. When the criterion of exchangeability between the training and test set is satisfied, the prediction error rate overlaps the selected significance level, which is one of the major strengths of this method. We assessed how the sensitivity is influenced by the choice of training set for two targets ($A_{2A}R$ and D_2R). A conformal predictor was trained on one million random molecules from WuXi's GalaXi make-on-demand database (1.4 billion rule-of-four molecules), which has only a small overlap with the Enamine's REAL

database.[14] Predictions were then performed for the set of ten million random molecules from Enamine's REAL database docked to the corresponding target. For both targets, substantially worse sensitivity values (0.19 and 0.30, respectively) were obtained compared to the scenario in which both the training and test set were randomly extracted from the Enamine's REAL database (0.89 and 0.92, respectively) (Supplementary Figure 12). This demonstrated that full exchangeability between training and test set is essential for accurate predictions.

Supplementary Table 2. Model hyperparameters. Key hyperparameters used during training of models.

Architecture	CatBoost	DNN	RoBERTa
Parameters	• nr_trees = 500<br>• metric = AUC<br>• weights = balanced	• learning_rate = 1e-4<br>• weight_decay = 1e-2<br>• batch_size = 200<br>• max_epochs= 100<br>• patience = 10<br>• optimizer = RangerLars<br>• class_weights = balanced	• learning_rate = 4e-7<br>• max_epochs = 10<br>• seyonec/PubChem10M

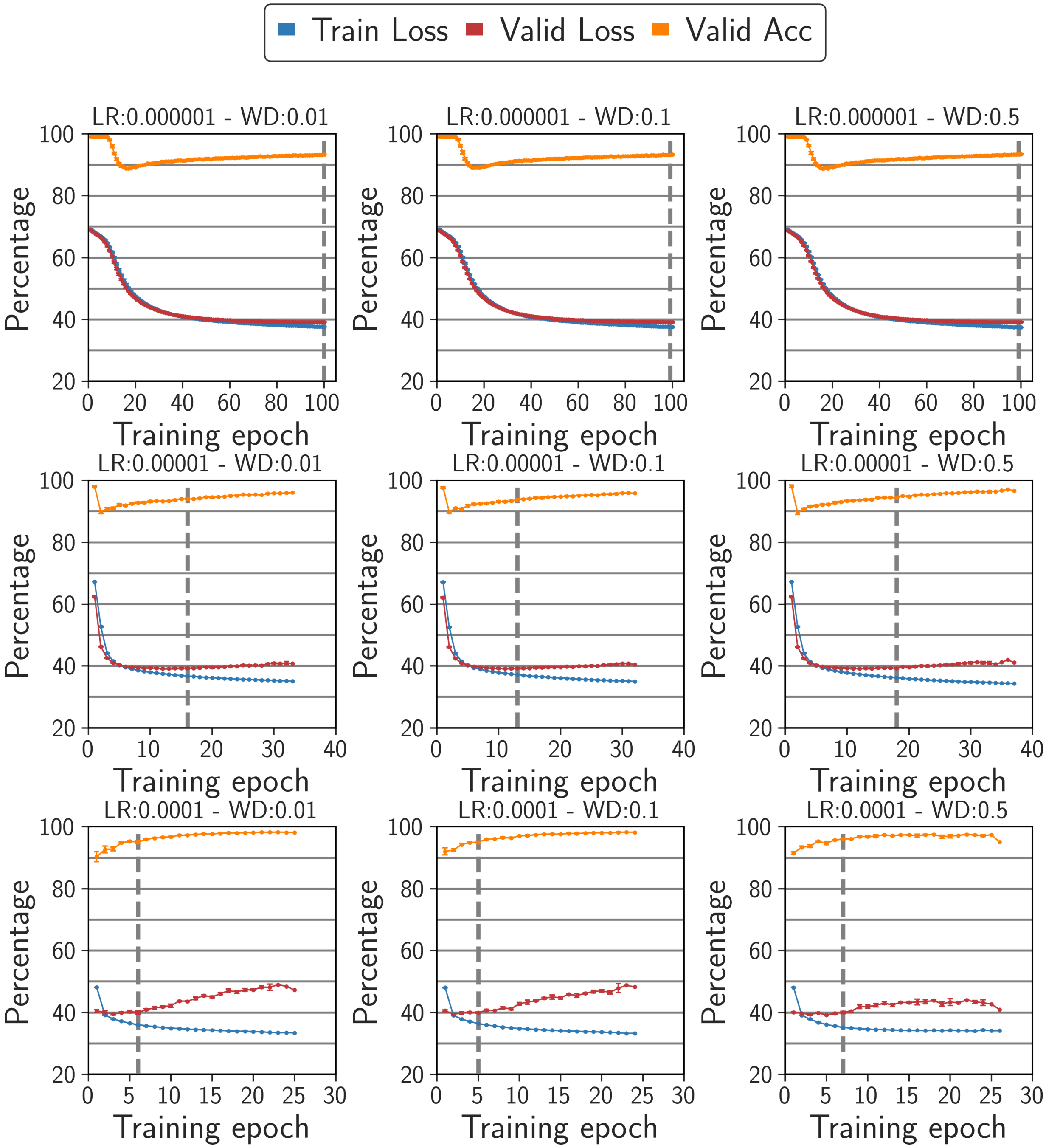

Supplementary Figure 3. Learning rate and weight decay analysis for deep neural networks. The changes in training loss, valid loss, valid accuracy, and speeds during training were monitored for deep neural networks with learning rates (LR) and weight decays (WD). Models were trained on one million molecules of the AmpC dataset represented by Morgan2 descriptors, and hence the input dimension was set to 1024. The output dimension was set to two for binary classification (virtual active and virtual inactive). The early stop patience for valid loss was set to 3, after which the best performing checkpoint (grey dashed line) was stored as final model. The default learning rate was then set to 1e-4 and the default weight decay was set to 1e-2 (See Supp Table S2). Mean values were obtained from three independently trained models and error bars correspond to the standard error of those means.

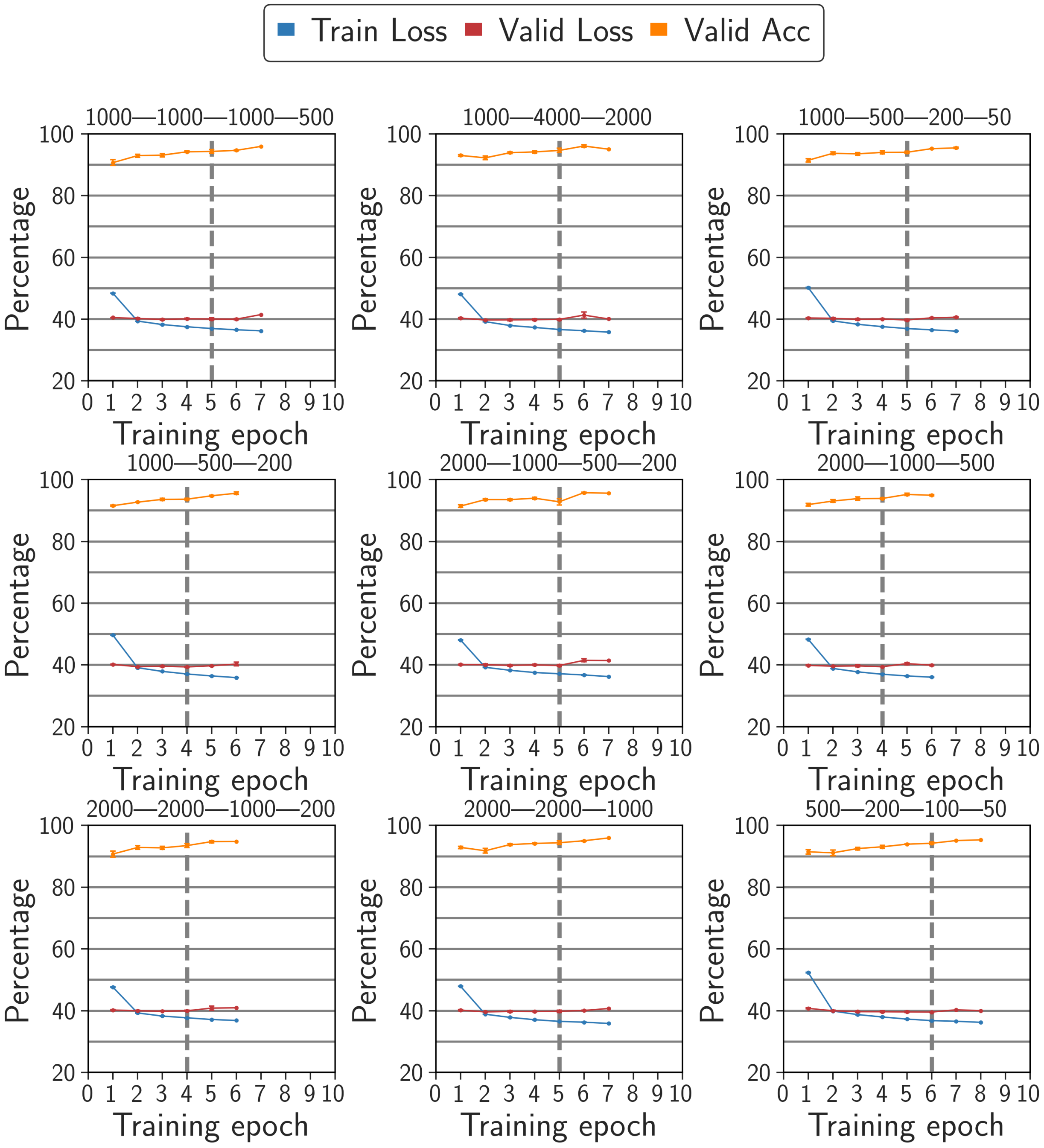

Supplementary Figure 4. Architecture analysis for deep neural networks. The changes in training loss, valid loss, and valid accuracy during training were monitored for deep neural networks with different architectures, which are shown above each subplot. Models were trained on one million molecules of the AmpC dataset represented by Morgan2 descriptors, and hence the input dimension was set to 1024. The output dimension was set to two for binary classification (virtual active and virtual inactive). The learning rate was set to 1e-4 and the weight decay was set to 1e-2. The early stop patience for valid loss was set to 3, after which the best performing checkpoint (grey dashed line) was stored as final model. The [input]-[1000]-[4000]-[2000]-[2] architecture was then selected as default. Mean values were obtained from three independently trained models and error bars correspond to the standard error of those means.

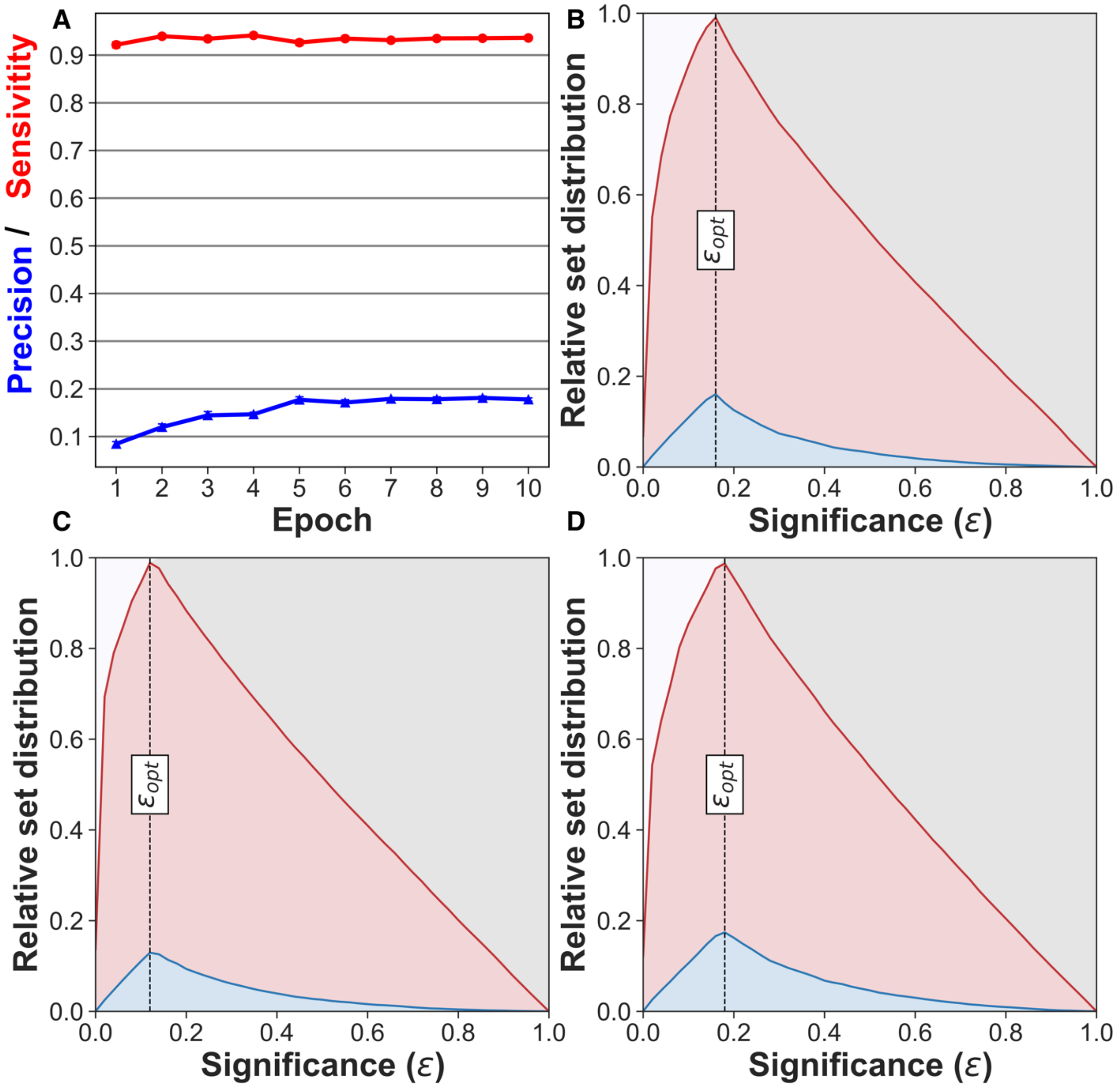

Supplementary Figure 5. Learning rate analysis for RoBERTa. (A) The changes in sensitivity and precision during training were monitored for the RoBERTa classifiers. Models were trained on one million AmpC molecules using RoBERTa's internal descriptors. A small external test set of 200000 molecules was used to obtain the sensitivity and precision metrics. Mean values were obtained from three independently trained models and predictions, and error bars correspond to the standard error of those means. The default number of epochs was set to ten in all other calculations. (B) RoBERTa models were trained on one million $A_{2A}R$ molecules with three different learning rates: 1e-5 (B), 4e-6 (C), and 4e-8 (D). The relative set distributions for different significance values are shown, together with the significance at which the predict achieves highest efficiency. The default learning rate was then set to 4e-7 for training RoBERTa models (See Supp Table S2). The fraction of molecules predicted to be in the one-set, zero-set, both-set, and null-set are colored in blue, red, white, and gray respectively (B,C,D).

Supplementary Table 3. Sensitivity and training set size - Morgan2. Sensitivity values obtained at optimal efficiency for different sizes of the training set.

Method	Target	Sensitivity[a]					
		25K	50K	100K	200K	500K	1M
CatBoost	$A_{2A}R$	0.754 ± 0.011	0.799 ± 0.005	0.820 ± 0.007	0.856 ± 0.004	0.873 ± 0.003	0.891 ± 0.002
	AmpC	0.857 ± 0.014	0.909 ± 0.009	0.921 ± 0.005	0.936 ± 0.001	0.945 ± 0.000	0.955 ± 0.001
	5'-NT	0.719 ± 0.025	0.773 ± 0.005	0.783 ± 0.008	0.811 ± 0.002	0.834 ± 0.001	0.849 ± 0.001
	D_2R	0.793 ± 0.002	0.813 ± 0.016	0.854 ± 0.006	0.883 ± 0.002	0.910 ± 0.001	0.917 ± 0.001
	KEAP1	0.688 ± 0.011	0.732 ± 0.008	0.777 ± 0.008	0.795 ± 0.005	0.819 ± 0.002	0.833 ± 0.003
	M^{PRO}	0.588 ± 0.010	0.650 ± 0.003	0.681 ± 0.003	0.705 ± 0.006	0.743 ± 0.003	0.765 ± 0.005
	OGG1	0.720 ± 0.014	0.770 ± 0.004	0.782 ± 0.001	0.815 ± 0.002	0.836 ± 0.006	0.853 ± 0.001
	SORT1	0.656 ± 0.011	0.703 ± 0.004	0.733 ± 0.003	0.773 ± 0.001	0.804 ± 0.004	0.821 ± 0.004
	**Average**	**0.722 ± 0.017**	**0.768 ± 0.015**	**0.794 ± 0.014**	**0.822 ± 0.014**	**0.845 ± 0.012**	**0.860 ± 0.012**
DNN	$A_{2A}R$	0.744 ± 0.034	0.789 ± 0.010	0.814 ± 0.013	0.833 ± 0.008	0.831 ± 0.007	0.841 ± 0.002
	AmpC	0.781 ± 0.013	0.836 ± 0.003	0.859 ± 0.004	0.897 ± 0.004	0.903 ± 0.001	0.919 ± 0.003
	5'-NT	0.731 ± 0.009	0.753 ± 0.018	0.782 ± 0.016	0.788 ± 0.010	0.807 ± 0.002	0.804 ± 0.002
	D_2R	0.747 ± 0.005	0.769 ± 0.012	0.803 ± 0.009	0.838 ± 0.005	0.861 ± 0.003	0.873 ± 0.003
	KEAP1	0.697 ± 0.035	0.766 ± 0.011	0.768 ± 0.014	0.784 ± 0.004	0.790 ± 0.004	0.796 ± 0.006
	M^{PRO}	0.677 ± 0.045	0.675 ± 0.014	0.682 ± 0.007	0.699 ± 0.003	0.713 ± 0.006	0.726 ± 0.002
	OGG1	0.728 ± 0.030	0.772 ± 0.012	0.787 ± 0.012	0.791 ± 0.006	0.806 ± 0.002	0.817 ± 0.002
	SORT1	0.704 ± 0.025	0.702 ± 0.007	0.729 ± 0.010	0.749 ± 0.010	0.760 ± 0.002	0.782 ± 0.004
	**Average**	**0.726 ± 0.010**	**0.758 ± 0.010**	**0.778 ± 0.011**	**0.797 ± 0.012**	**0.809 ± 0.012**	**0.820 ± 0.011**

[a] Each test set contained ten million molecules. Morgan2 descriptors were used as features of the molecules. Three independent calculations (training and prediction) were performed for each target and error bars correspond to the standard error of the mean. Averages are reported in bold.

Supplementary Table 4. Precision and training set size - Morgan2. Precision values obtained at optimal efficiency for different sizes of the training set.

Method	Target	Precision[a]					
		25K	50K	100K	200K	500K	1M
CatBoost	$A_{2A}R$	0.043 ± 0.002	0.045 ± 0.001	0.052 ± 0.002	0.055 ± 0.000	0.068 ± 0.001	0.074 ± 0.001
	AmpC	0.090 ± 0.003	0.100 ± 0.005	0.117 ± 0.002	0.138 ± 0.007	0.180 ± 0.002	0.202 ± 0.001
	5'-NT	0.035 ± 0.002	0.036 ± 0.001	0.039 ± 0.001	0.041 ± 0.000	0.046 ± 0.000	0.052 ± 0.000
	D_2R	0.047 ± 0.000	0.060 ± 0.005	0.065 ± 0.003	0.079 ± 0.002	0.093 ± 0.000	0.106 ± 0.000
	KEAP1	0.034 ± 0.001	0.034 ± 0.001	0.036 ± 0.000	0.040 ± 0.000	0.044 ± 0.000	0.047 ± 0.001
	M^{PRO}	0.020 ± 0.000	0.022 ± 0.000	0.023 ± 0.000	0.025 ± 0.000	0.028 ± 0.000	0.030 ± 0.000
	OGG1	0.035 ± 0.001	0.037 ± 0.001	0.040 ± 0.001	0.042 ± 0.000	0.048 ± 0.001	0.052 ± 0.000
	SORT1	0.024 ± 0.001	0.027 ± 0.000	0.030 ± 0.001	0.033 ± 0.001	0.039 ± 0.001	0.044 ± 0.001
	**Average**	**0.041 ± 0.004**	**0.045 ± 0.005**	**0.050 ± 0.006**	**0.057 ± 0.007**	**0.068 ± 0.010**	**0.076 ± 0.011**
DNN	$A_{2A}R$	0.039 ± 0.003	0.041 ± 0.001	0.044 ± 0.002	0.048 ± 0.001	0.054 ± 0.001	0.057 ± 0.000
	AmpC	0.042 ± 0.002	0.052 ± 0.002	0.067 ± 0.002	0.080 ± 0.003	0.097 ± 0.002	0.104 ± 0.001
	5'-NT	0.030 ± 0.001	0.034 ± 0.001	0.035 ± 0.001	0.039 ± 0.001	0.041 ± 0.001	0.043 ± 0.000
	D_2R	0.029 ± 0.001	0.040 ± 0.003	0.045 ± 0.003	0.053 ± 0.002	0.060 ± 0.001	0.068 ± 0.001
	KEAP1	0.029 ± 0.002	0.030 ± 0.001	0.034 ± 0.001	0.035 ± 0.001	0.039 ± 0.001	0.040 ± 0.001
	M^{PRO}	0.018 ± 0.001	0.021 ± 0.000	0.023 ± 0.000	0.024 ± 0.000	0.026 ± 0.000	0.027 ± 0.000
	OGG1	0.034 ± 0.001	0.037 ± 0.001	0.038 ± 0.001	0.041 ± 0.001	0.043 ± 0.000	0.044 ± 0.000
	SORT1	0.022 ± 0.001	0.025 ± 0.001	0.028 ± 0.001	0.030 ± 0.001	0.033 ± 0.000	0.035 ± 0.000
	**Average**	**0.031 ± 0.002**	**0.035 ± 0.002**	**0.039 ± 0.003**	**0.044 ± 0.003**	**0.049 ± 0.004**	**0.052 ± 0.005**

[a] Each test set contained ten million molecules. Morgan2 descriptors were used as features of the molecules. Three independent calculations (training and prediction) were performed for each target and error bars correspond to the standard error of the mean. Averages are reported in bold.

Supplementary Table 5. Sensitivity and training set size - CDDD. Sensitivity values obtained at optimal efficiency for different sizes of the training set.

Method	Target	Sensitivity[a]					
		25K	50K	100K	200K	500K	1M
CatBoost	$A_{2A}R$	0.784 ± 0.013	0.806 ± 0.013	0.819 ± 0.003	0.845 ± 0.002	0.852 ± 0.003	0.870 ± 0.004
	AmpC	0.847 ± 0.013	0.893 ± 0.008	0.903 ± 0.004	0.919 ± 0.003	0.931 ± 0.001	0.937 ± 0.002
	5'-NT	0.747 ± 0.012	0.790 ± 0.007	0.793 ± 0.005	0.815 ± 0.004	0.828 ± 0.003	0.832 ± 0.002
	D_2R	0.805 ± 0.014	0.839 ± 0.004	0.847 ± 0.008	0.875 ± 0.001	0.888 ± 0.001	0.896 ± 0.002
	KEAP1	0.716 ± 0.014	0.759 ± 0.008	0.784 ± 0.009	0.799 ± 0.002	0.816 ± 0.003	0.827 ± 0.001
	M^{PRO}	0.605 ± 0.004	0.658 ± 0.004	0.682 ± 0.003	0.699 ± 0.005	0.728 ± 0.003	0.737 ± 0.007
	OGG1	0.745 ± 0.007	0.776 ± 0.005	0.776 ± 0.006	0.809 ± 0.001	0.816 ± 0.002	0.833 ± 0.003
	SORT1	0.676 ± 0.006	0.691 ± 0.011	0.722 ± 0.004	0.749 ± 0.005	0.772 ± 0.004	0.792 ± 0.001
	**Average**	**0.741 ± 0.015**	**0.777 ± 0.015**	**0.791 ± 0.014**	**0.814 ± 0.014**	**0.829 ± 0.012**	**0.840 ± 0.012**
DNN	$A_{2A}R$	0.755 ± 0.005	0.832 ± 0.013	0.818 ± 0.008	0.851 ± 0.004	0.850 ± 0.003	0.862 ± 0.002
	AmpC	0.841 ± 0.016	0.871 ± 0.011	0.892 ± 0.007	0.908 ± 0.005	0.923 ± 0.004	0.941 ± 0.004
	5'-NT	0.777 ± 0.016	0.812 ± 0.000	0.816 ± 0.015	0.811 ± 0.007	0.822 ± 0.002	0.836 ± 0.003
	D_2R	0.786 ± 0.021	0.874 ± 0.005	0.857 ± 0.006	0.871 ± 0.002	0.884 ± 0.003	0.897 ± 0.003
	KEAP1	0.753 ± 0.022	0.792 ± 0.015	0.791 ± 0.008	0.810 ± 0.007	0.824 ± 0.005	0.820 ± 0.001
	M^{PRO}	0.657 ± 0.025	0.707 ± 0.004	0.689 ± 0.013	0.724 ± 0.002	0.723 ± 0.008	0.737 ± 0.006
	OGG1	0.772 ± 0.017	0.798 ± 0.003	0.800 ± 0.007	0.802 ± 0.014	0.821 ± 0.005	0.828 ± 0.002
	SORT1	0.684 ± 0.042	0.728 ± 0.009	0.761 ± 0.003	0.772 ± 0.012	0.775 ± 0.003	0.793 ± 0.006
	**Average**	**0.753 ± 0.013**	**0.802 ± 0.012**	**0.803 ± 0.012**	**0.819 ± 0.012**	**0.828 ± 0.012**	**0.839 ± 0.012**

[a] Each test set contained ten million molecules. Continuous-Data-Driven Descriptors (CDDD) were used as features of the molecules. Three independent calculations (training and prediction) were performed for each target and error bars correspond to the standard error of the mean. Averages are reported in bold.

Supplementary Table 6. Precision and training set size - CDDD. Precision values obtained at optimal efficiency for different sizes of the training set.

Method	Target	Precision[a]					
		25K	50K	100K	200K	500K	1M
CatBoost	$A_{2A}R$	0.046 ± 0.002	0.047 ± 0.001	0.051 ± 0.001	0.052 ± 0.001	0.059 ± 0.000	0.062 ± 0.000
	AmpC	0.079 ± 0.001	0.085 ± 0.003	0.093 ± 0.000	0.100 ± 0.002	0.113 ± 0.001	0.128 ± 0.002
	5'-NT	0.042 ± 0.002	0.039 ± 0.001	0.044 ± 0.001	0.043 ± 0.001	0.046 ± 0.000	0.050 ± 0.000
	D_2R	0.052 ± 0.002	0.057 ± 0.001	0.060 ± 0.003	0.063 ± 0.000	0.071 ± 0.000	0.079 ± 0.001
	KEAP1	0.038 ± 0.001	0.037 ± 0.001	0.038 ± 0.001	0.040 ± 0.000	0.042 ± 0.000	0.045 ± 0.000
	M^{PRO}	0.021 ± 0.000	0.022 ± 0.001	0.024 ± 0.000	0.025 ± 0.000	0.026 ± 0.000	0.027 ± 0.000
	OGG1	0.035 ± 0.000	0.038 ± 0.000	0.041 ± 0.000	0.041 ± 0.000	0.044 ± 0.000	0.046 ± 0.000
	SORT1	0.026 ± 0.000	0.027 ± 0.001	0.028 ± 0.000	0.030 ± 0.000	0.034 ± 0.001	0.037 ± 0.000
	**Average**	**0.042 ± 0.003**	**0.044 ± 0.004**	**0.047 ± 0.004**	**0.049 ± 0.005**	**0.054 ± 0.005**	**0.059 ± 0.006**
DNN	$A_{2A}R$	0.054 ± 0.002	0.047 ± 0.001	0.056 ± 0.000	0.053 ± 0.001	0.064 ± 0.001	0.067 ± 0.001
	AmpC	0.089 ± 0.004	0.101 ± 0.002	0.105 ± 0.003	0.126 ± 0.005	0.135 ± 0.002	0.140 ± 0.003
	5'-NT	0.042 ± 0.002	0.041 ± 0.001	0.043 ± 0.002	0.047 ± 0.001	0.049 ± 0.000	0.050 ± 0.000
	D_2R	0.062 ± 0.004	0.057 ± 0.001	0.068 ± 0.001	0.071 ± 0.002	0.079 ± 0.001	0.084 ± 0.001
	KEAP1	0.037 ± 0.001	0.038 ± 0.002	0.042 ± 0.000	0.044 ± 0.001	0.045 ± 0.001	0.049 ± 0.000
	M^{PRO}	0.023 ± 0.001	0.023 ± 0.000	0.025 ± 0.001	0.026 ± 0.000	0.028 ± 0.000	0.029 ± 0.000
	OGG1	0.038 ± 0.001	0.040 ± 0.001	0.042 ± 0.001	0.045 ± 0.001	0.048 ± 0.000	0.050 ± 0.000
	SORT1	0.029 ± 0.002	0.029 ± 0.000	0.030 ± 0.001	0.034 ± 0.001	0.038 ± 0.001	0.040 ± 0.001
	**Average**	**0.047 ± 0.004**	**0.047 ± 0.005**	**0.051 ± 0.005**	**0.055 ± 0.006**	**0.061 ± 0.007**	**0.064 ± 0.007**

[a] Each test set contained ten million molecules. Continuous-Data-Driven Descriptors (CDDD) were used as features of the molecules. Three independent calculations (training and prediction) were performed for each target and error bars correspond to the standard error of the mean. Averages are reported in bold.

Supplementary Table 7. Sensitivity and training set size - RoBERTa. Sensitivity values obtained at optimal efficiency for different sizes of the training set.

Method	Target	Sensitivity[a]					
		25K	50K	100K	200K	500K	1M
RoBERTa	$A_{2A}R$	0.765 ± 0.007	0.781 ± 0.006	0.806 ± 0.007	0.848 ± 0.007	0.861 ± 0.006	0.879 ± 0.002
	AmpC	0.808 ± 0.005	0.872 ± 0.005	0.890 ± 0.004	0.916 ± 0.003	0.939 ± 0.002	0.944 ± 0.002
	5'-NT	0.735 ± 0.011	0.784 ± 0.007	0.778 ± 0.005	0.808 ± 0.007	0.827 ± 0.003	0.841 ± 0.003
	D_2R	0.737 ± 0.003	0.817 ± 0.004	0.841 ± 0.007	0.863 ± 0.002	0.884 ± 0.003	0.901 ± 0.000
	KEAP1	0.727 ± 0.011	0.764 ± 0.006	0.797 ± 0.005	0.805 ± 0.006	0.822 ± 0.005	0.830 ± 0.000
	M^{PRO}	0.627 ± 0.005	0.657 ± 0.013	0.689 ± 0.005	0.703 ± 0.002	0.729 ± 0.001	0.745 ± 0.000
	OGG1	0.728 ± 0.007	0.751 ± 0.005	0.783 ± 0.003	0.805 ± 0.004	0.819 ± 0.005	0.837 ± 0.004
	SORT1	0.662 ± 0.014	0.690 ± 0.010	0.730 ± 0.001	0.757 ± 0.003	0.782 ± 0.001	0.805 ± 0.004
	**Average**	**0.724 ± 0.011**	**0.764 ± 0.013**	**0.789 ± 0.012**	**0.813 ± 0.013**	**0.833 ± 0.012**	**0.848 ± 0.012**

[a] Each test set contained ten million molecules. Internal RoBERTa descriptors were used as features of the molecules. Three independent calculations (training and prediction) were performed for each target and error bars correspond to the standard error of the mean. Averages are reported in bold.

Supplementary Table 8. Precision and training set size - RoBERTa. Precision values obtained at optimal efficiency for different sizes of the training set.

Method	Target	Precision[a]					
		25K	50K	100K	200K	500K	1M
RoBERTa	$A_{2A}R$	0.034 ± 0.001	0.042 ± 0.001	0.050 ± 0.001	0.054 ± 0.001	0.064 ± 0.002	0.070 ± 0.000
	AmpC	0.052 ± 0.000	0.065 ± 0.001	0.084 ± 0.002	0.111 ± 0.002	0.143 ± 0.004	0.181 ± 0.004
	5'-NT	0.032 ± 0.001	0.035 ± 0.001	0.042 ± 0.000	0.045 ± 0.001	0.050 ± 0.001	0.054 ± 0.001
	D_2R	0.034 ± 0.001	0.048 ± 0.001	0.058 ± 0.001	0.066 ± 0.002	0.082 ± 0.001	0.094 ± 0.001
	KEAP1	0.035 ± 0.001	0.038 ± 0.001	0.040 ± 0.000	0.043 ± 0.001	0.047 ± 0.001	0.050 ± 0.000
	M^{PRO}	0.018 ± 0.000	0.021 ± 0.000	0.023 ± 0.000	0.025 ± 0.000	0.028 ± 0.000	0.031 ± 0.000
	OGG1	0.030 ± 0.000	0.035 ± 0.000	0.039 ± 0.000	0.042 ± 0.001	0.048 ± 0.001	0.051 ± 0.000
	SORT1	0.022 ± 0.001	0.026 ± 0.000	0.029 ± 0.000	0.031 ± 0.000	0.038 ± 0.000	0.043 ± 0.000
	**Average**	**0.032 ± 0.002**	**0.039 ± 0.003**	**0.046 ± 0.004**	**0.052 ± 0.005**	**0.062 ± 0.007**	**0.072 ± 0.009**

[a] Each test set contained ten million molecules. Internal RoBERTa descriptors were used as features of the molecules. Three independent calculations (training and prediction) were performed for each target and error bars correspond to the standard error of the mean. Averages are reported in bold.

Supplementary Table 9. **Training and prediction times**.

Cost (s)[a]	CatBoost		DNN		RoBERTa (GPU)
Descriptor	Morgan2	CDDD	Morgan2	CDDD	
Training (1M)	1410	2330	12669	24906	376685
Prediction (1M)	117	192	462	699	9125

[a] The times (in seconds) required to train a conformal predictor on one million molecules with different architectures and descriptors or predict one million molecules.

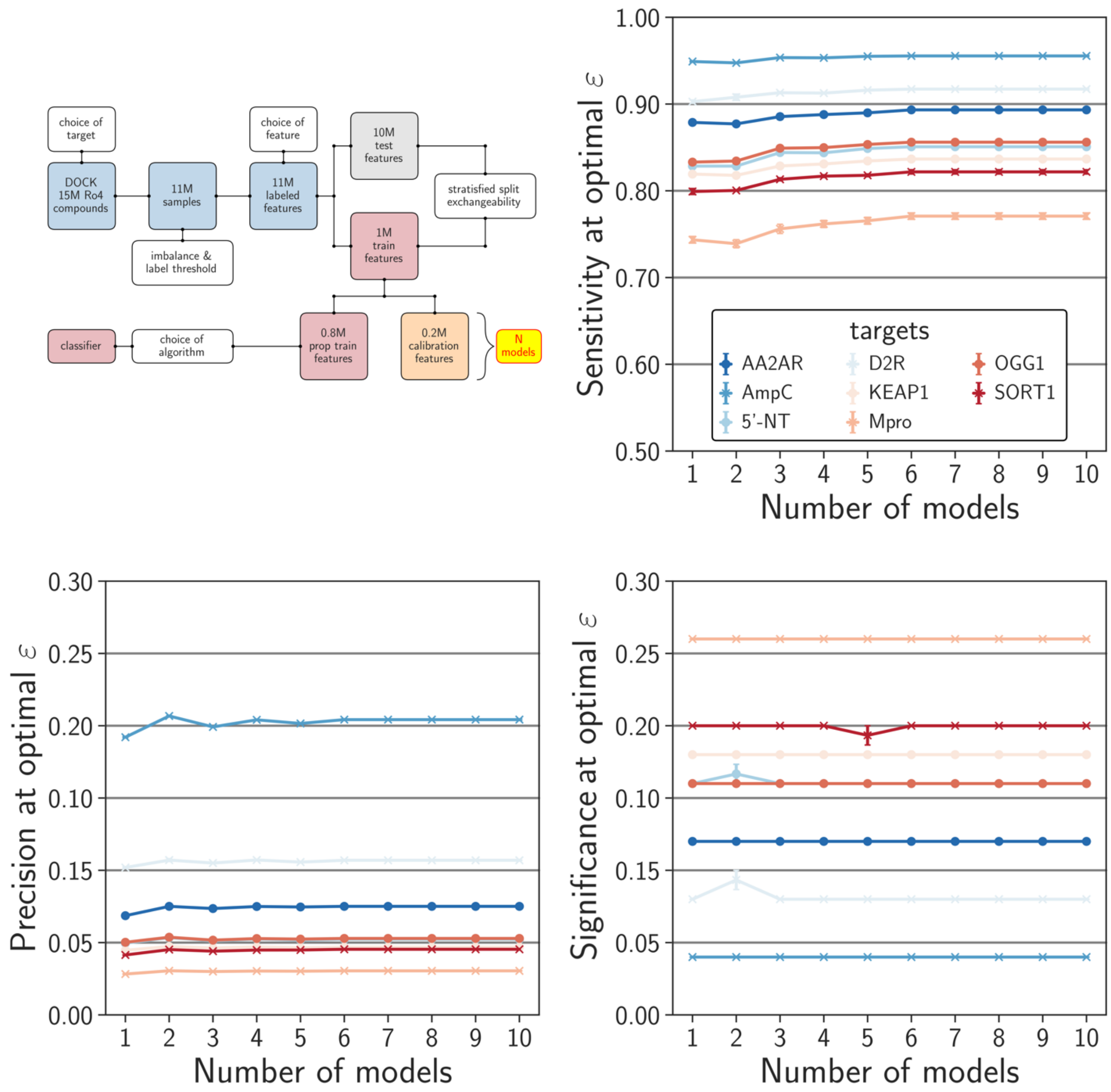

Supplementary Figure 6. Performance and number of aggregated models. Sensitivity and precision at optimal efficiency were analyzed for a different number of models during aggregation. Five independent CatBoost models were trained on one million molecules represented by Morgan2 descriptors. Each test set contained ten million molecules. Three independent calculations (training and prediction) were performed for the eight targets and error bars correspond to the standard error of the mean.

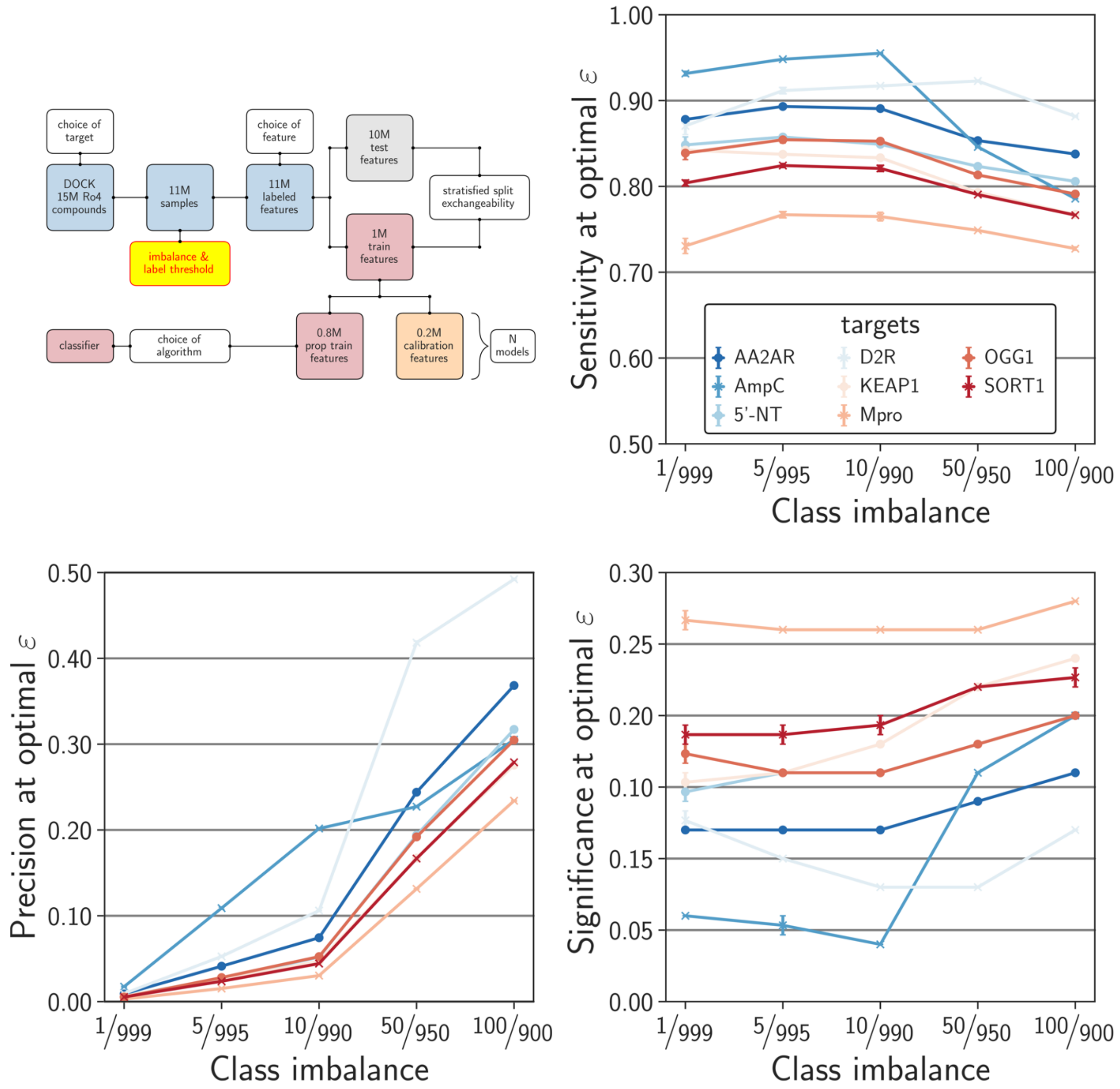

Supplementary Figure 7. Performance on imbalanced datasets. Sensitivity and precision at optimal efficiency were analyzed for different class imbalances. Five independent CatBoost models were trained on one million molecules represented by Morgan2 descriptors. Each test set contained ten million molecules. Three independent calculations (training and prediction) were performed for the eight targets and error bars correspond to the standard error of the mean.

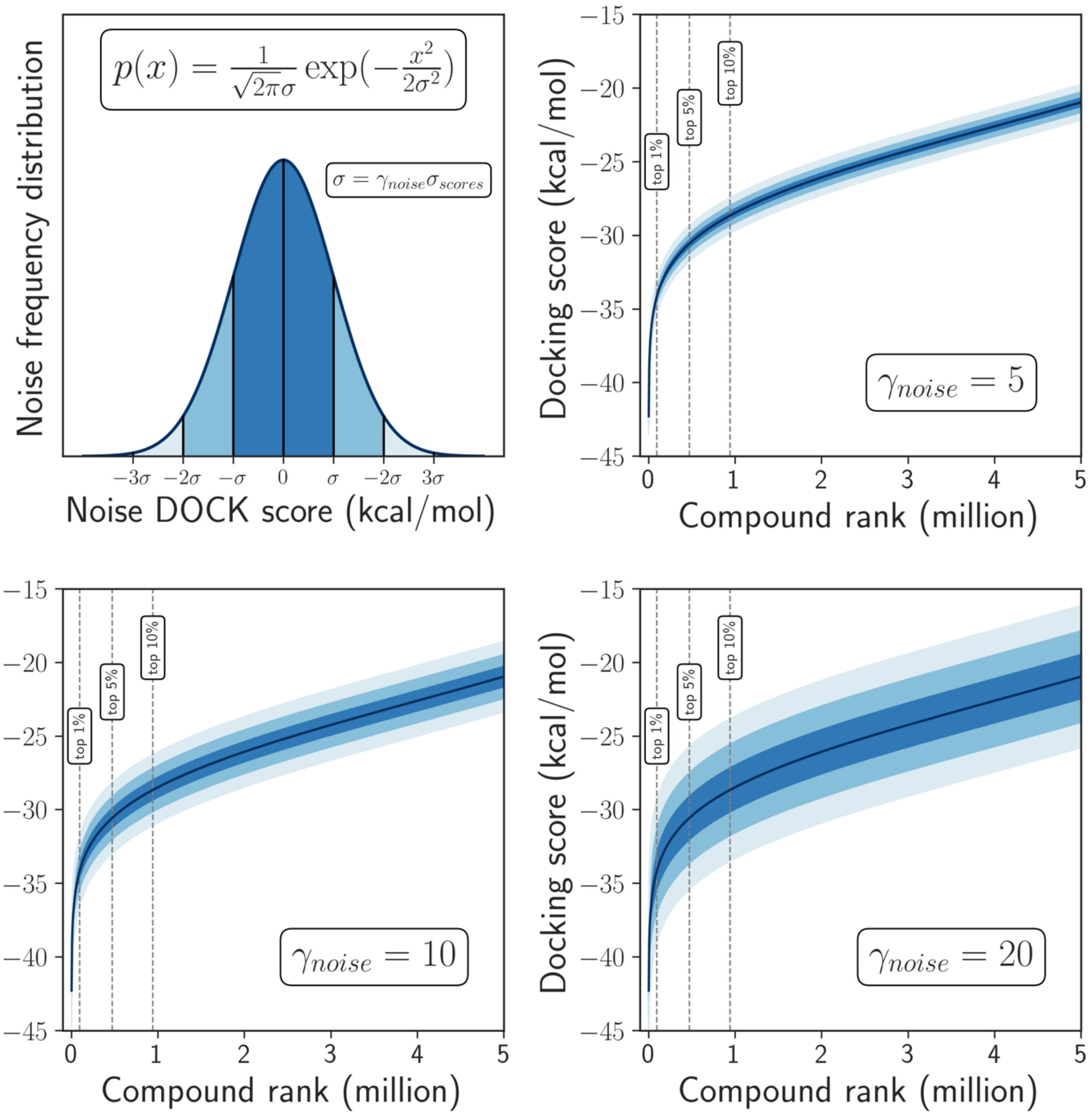

Supplementary Figure 8. Overview of noise addition. A zero-centered normal distribution was constructed using the standard deviation (σ_{scores}) of the docking score distribution and a noise scaling factor (γ_{noise}). Noise was added to the score of each sample by taking a sample from the corresponding noise distribution. Large noise scaling factors led to wide distributions and increased perturbations of the initial docking score distributions.

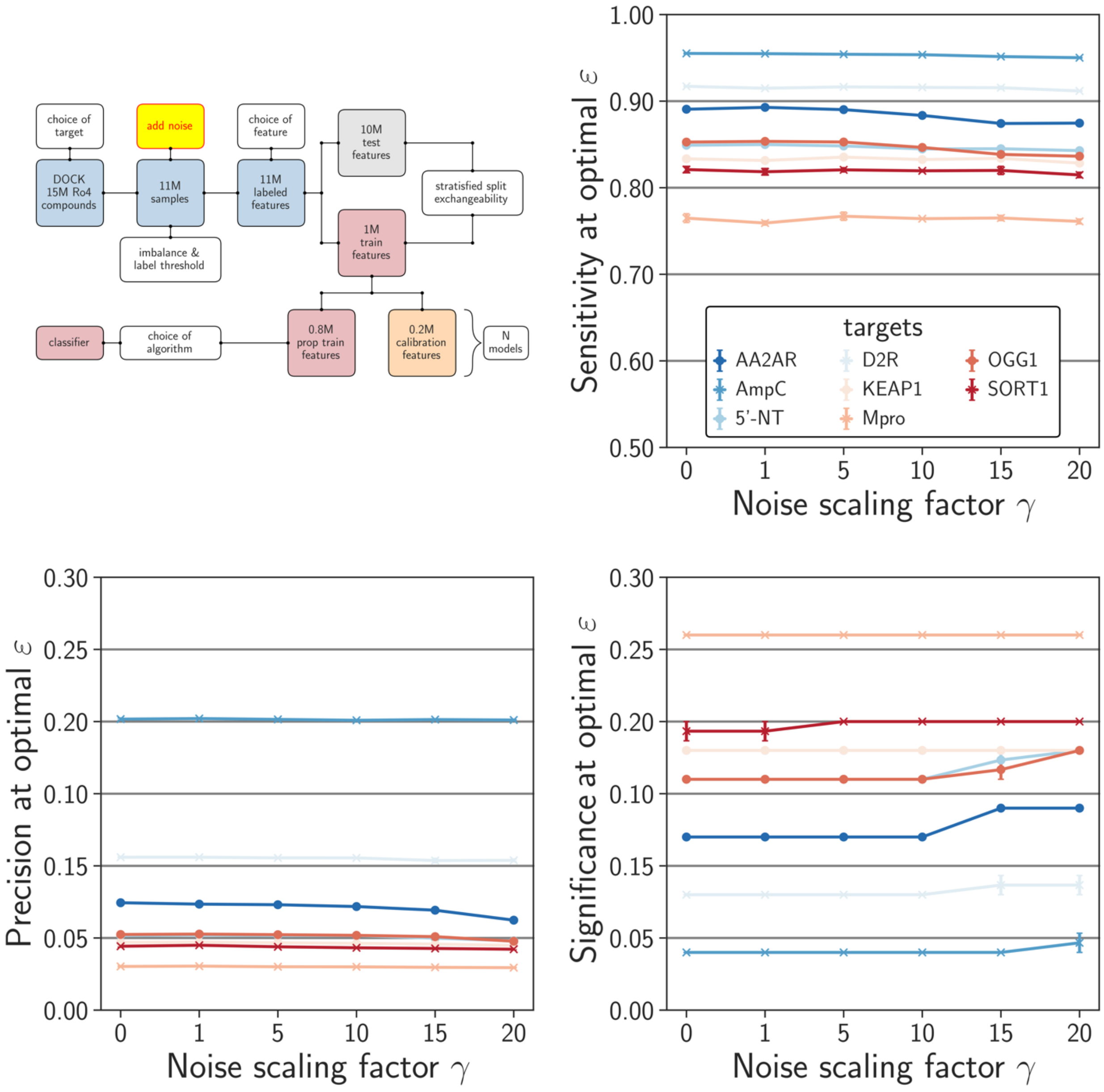

Supplementary Figure 9. Performance on noisy datasets. Sensitivity and precision at optimal efficiency were analyzed for datasets generated with different noise scaling factors (γ_{noise}). Five independent CatBoost models were trained on one million molecules represented by Morgan2 descriptors. Each test set contained ten million molecules. Three independent calculations (training and prediction) were performed for the eight targets and error bars correspond to the standard error of the mean.

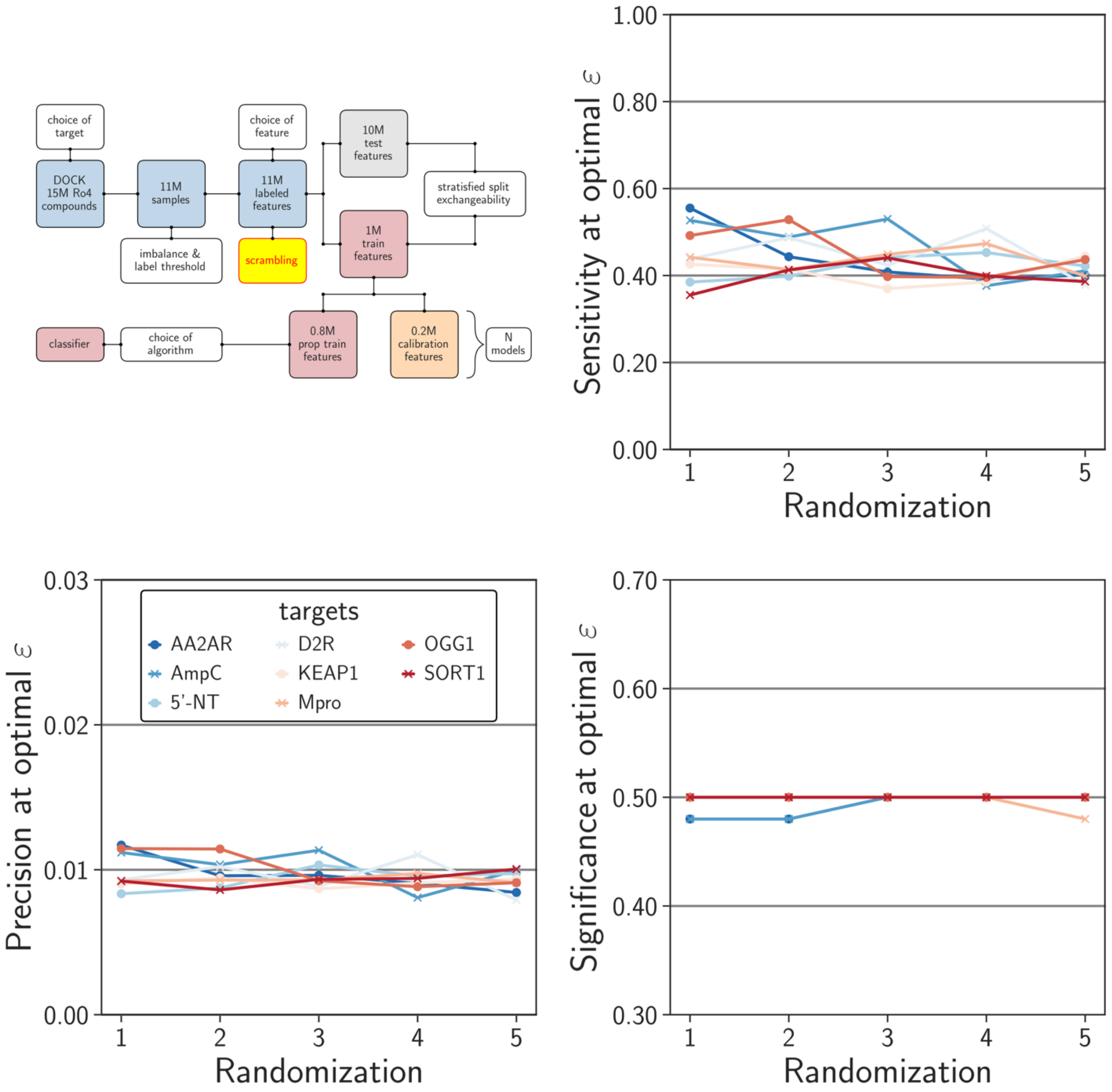

Supplementary Figure 10. Performance on non-sensical datasets - labels. Sensitivity and precision at optimal efficiency were analyzed for datasets where the labels were scrambled without affecting the class imbalance. Five independent CatBoost models were trained on one million molecules represented by Morgan2 descriptors. Each test set contained ten million molecules. Five independent calculations (training and prediction) were performed for the eight targets. When the CP operates at an optimal efficiency of 50%, has a sensitivity averaging around 50%, and a precision close to the class imbalance (1%), the performance will correspond to random classification. Values represent individual datapoints and no corresponding error bars are shown.

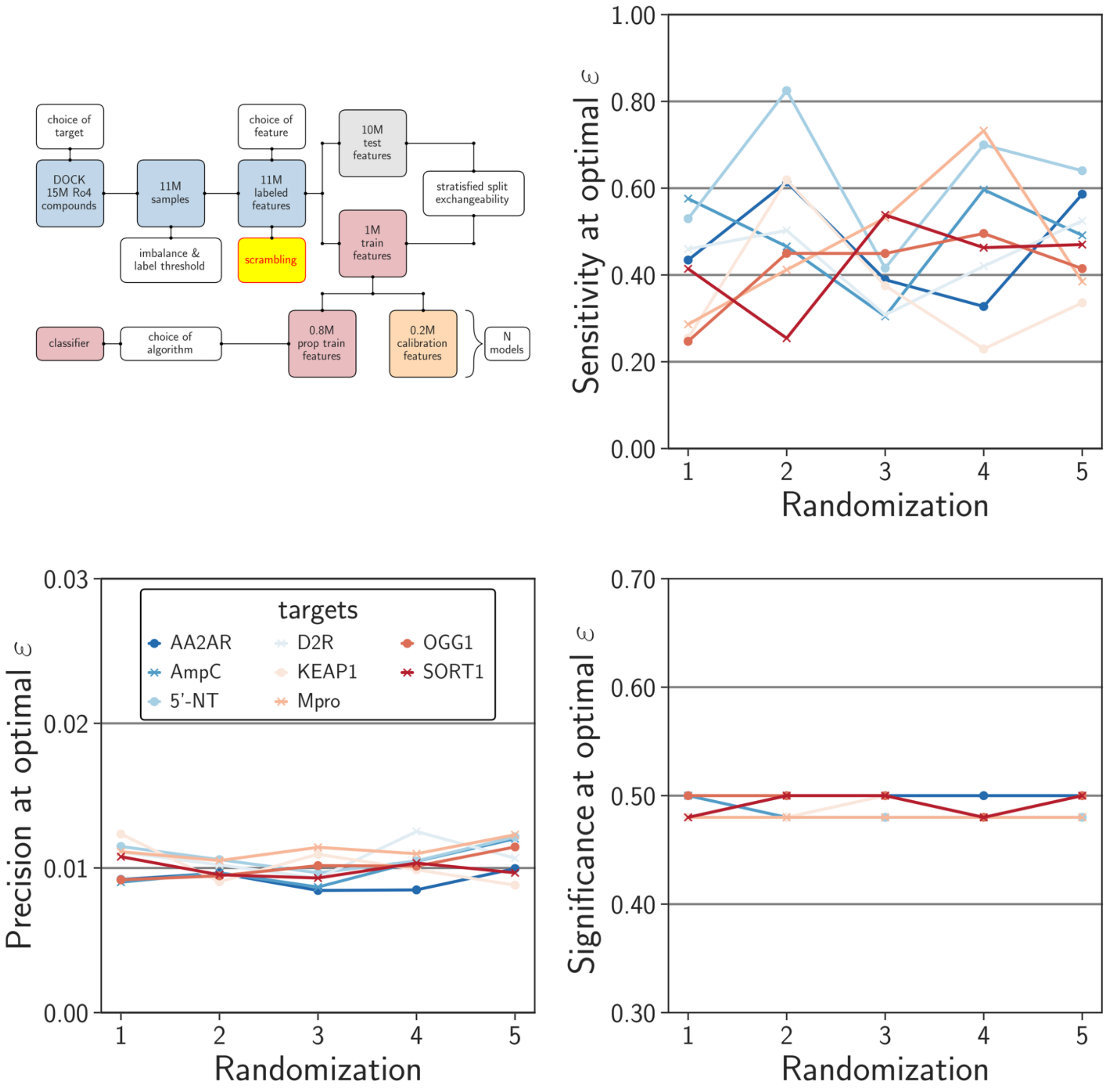

Supplementary Figure 11. Performance on non-sensical datasets - features. Sensitivity and precision at optimal efficiency were analyzed for datasets where the feature vectors were shuffled. Five independent CatBoost models were trained on one million molecules represented by Morgan2 descriptors. Each test set contained ten million molecules. Five independent calculations (training and prediction) were performed for the eight targets. When the CP operates at an optimal efficiency of 50%, has a sensitivity averaging around 50%, and a precision close to the class imbalance (1%), the performance will correspond to random classification. Values represent individual datapoints and no corresponding error bars are shown.

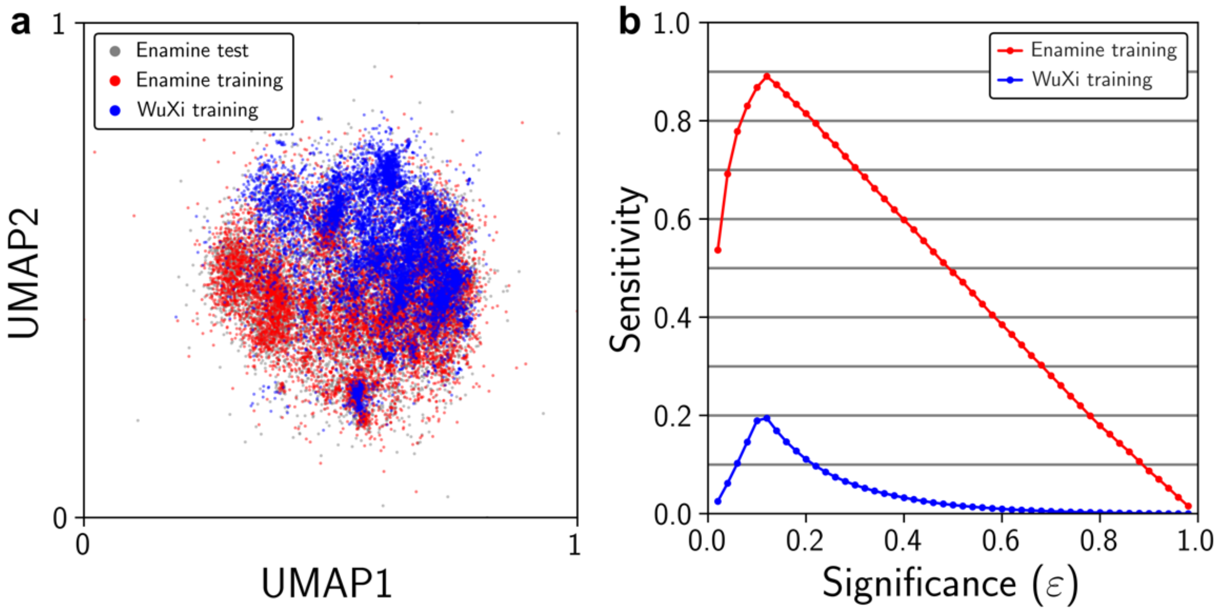

Supplementary Figure 12. Structural similarity between non-exchangeable datasets and conformal predictor performance. a) Two-dimensional unsupervised UMAP projection illustrates the chemical relationships in high-dimensional feature space between WuXi training set (blue), Enamine training set (red) and Enamine test (gray) sets (b) Difference in sensitivity values obtained from conformal predictors trained on one million exchangeable (red) and one million non-exchangeable (blue) molecules as a function of the significance value (ε). Values represent individual datapoints and no corresponding error bars are shown.

a

p_1 - p_0 Pearson correlation		
Target	Ranks	Scores
$A_{2A}R$	-0.590	-0.218
AmpC	-0.384	-0.072
D_2R	-0.481	-0.059
5'-NT	-0.634	-0.078
OGG1	-0.552	-0.098
SORT1	-0.494	-0.084
Mpro	-0.467	-0.087
KEAP1	-0.541	-0.079

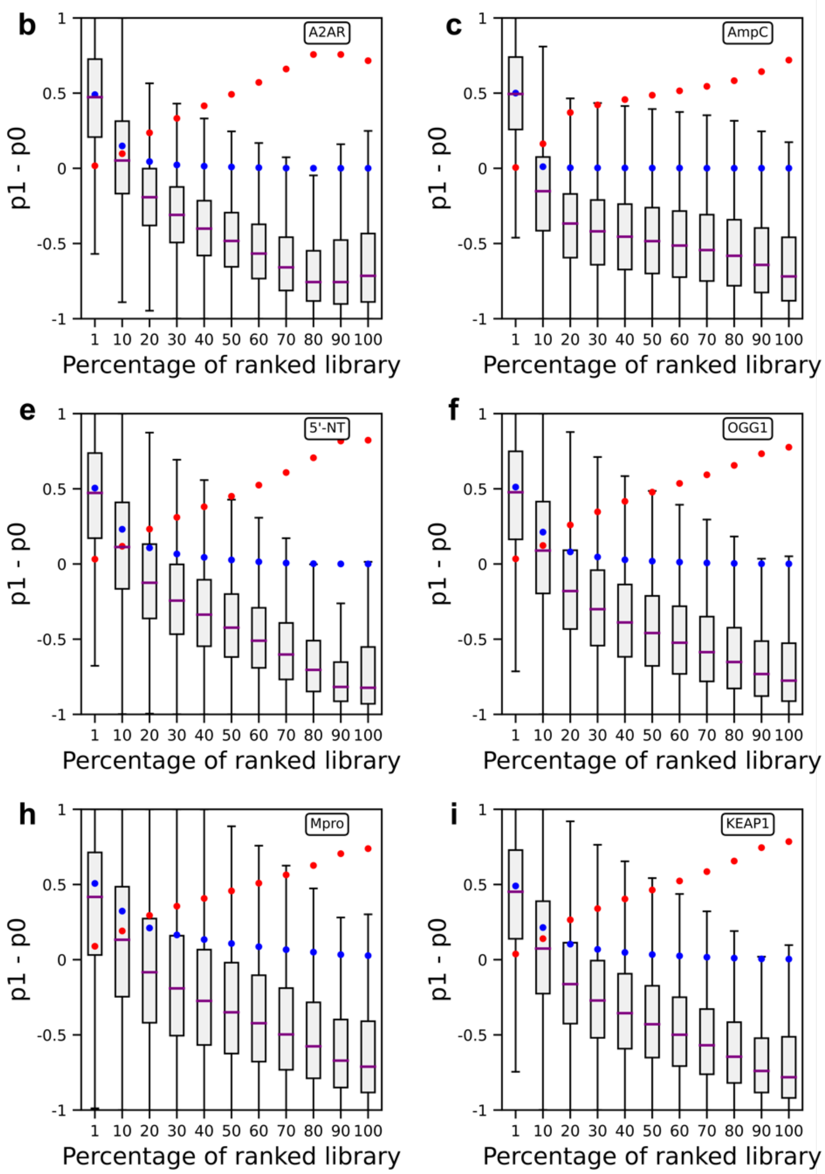

Supplementary Figure 13. Correlations between the quality of information metric and molecular docking results. (a) Pearson correlation coefficients between the quality of information metric (p_1 - p_0) and molecular docking results (ranks or scores) for eight different protein targets. (b-i) For eight different protein targets, boxplots representing the distribution of the quality of information metric (p_1 - p_0) across different segments of the library (ten million molecules) ranked by docking scores. Each box spans from the first quartile (Q1, 25th percentile) to the third quartile (Q3, 75th percentile), with the purple line inside the box indicating the median (50th percentile). The whiskers extend to the most extreme data points within 1.5 times the interquartile range (IQR) from the quartiles. Data points outside this range are considered outliers and are not visualized for clarity. Blue and red dots respectively represent the median p_1 and p_0 values for the different segments.

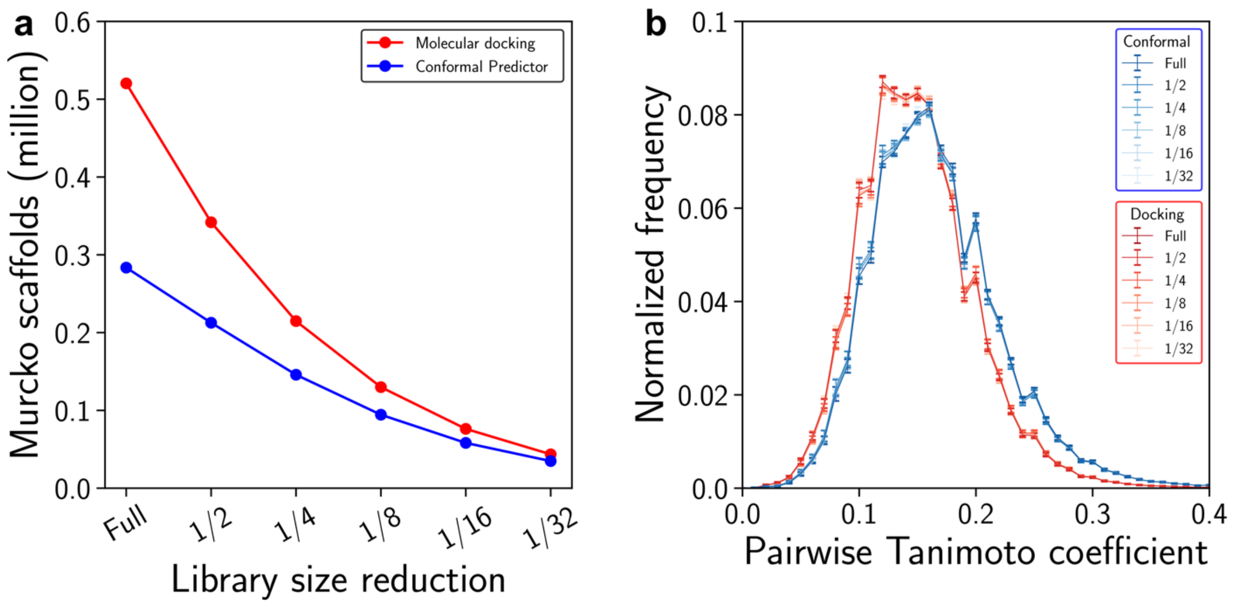

Supplementary Figure 14. (a) Number of unique Bemis-Murcko scaffolds in the top-ranked (1%) D_2R compounds prioritized by explicit docking (red) or the conformal predictor (blue) in function of the size of the virtual library. Values represent individual datapoints and no corresponding error bars are shown. (b) Distributions of pairwise Tanimoto coefficients in the top-ranked (1%) compounds prioritized by explicit docking (red) or the conformal predictor (blue) in function of the size of the virtual library. Ten random samples with no overlap were taken from the top-ranked (1%) compounds and their pairwise Tanimoto coefficients were calculated, followed by division of the results in one hundred bins. Data points represent the means of each bin, and error bars correspond to the standard errors on those means. A paired t-test indicates that the distributions of pairwise Tanimoto coefficients in the top-ranked (1%) compounds from the full library prioritized by explicit docking or the conformal predictor are not significantly different (p = 3.15e-6).

Supplementary Tables

Supplementary Table 10. Chemical structures and D_2R radioligand displacement data.

Chemical Structure	SMILES	Vendor code[a]	Displacement at 10 μM (%)[b]
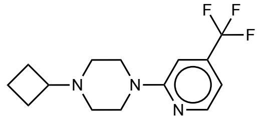	c1cnc(cc1C(F)(F)F)N2CCN(CC2)C3CCC3	Z1348398263	5 ± 3%
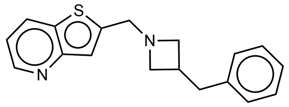	c1ccc(cc1)CC2CN(C2)Cc3cc4c(s3)cccn4	Z8185092667	12 ± 3%
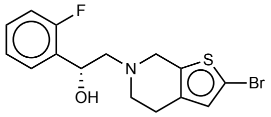	c1ccc(c(c1)[C@H](CN2CCc3cc(sc3C2)Br)O)F	Z8185092668	3 ± 1%
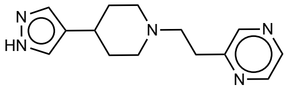	c1cnc(cn1)CCN2CCC(CC2)c3c[nH]nc3	Z2833584438	1 ± 1%
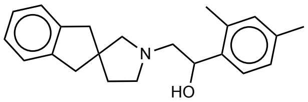	Cc1ccc(c(c1)C)C(CN2CCC3(C2)Cc4ccccc4C3)O	Z8185092353	4 ± 3%
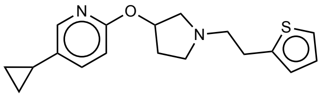	c1cc(sc1)CCN2CCC(C2)Oc3ccc(cn3)C4CC4	Z2694200197	17 ± 4%
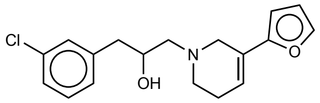	c1cc(cc(c1)Cl)CC(CN2CCC=C(C2)c3ccco3)O	Z2102774071	10 ± 3%
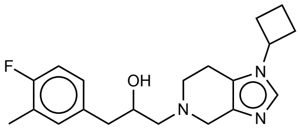	Cc1cc(ccc1F)CC(CN2CCc3c(ncn3C4CCC4)C2)O	Z3516919089	1 ± 1%
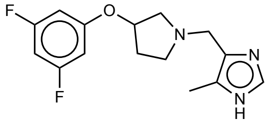	Cc1c(nc[nH]1)CN2CCC(C2)Oc3cc(cc(c3)F)F	Z3516846214	5 ± 1%
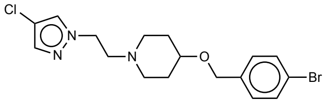	c1cc(ccc1COC2CCN(CC2)CCn3cc(cn3)Cl)Br	Z8185092665	4 ± 1%
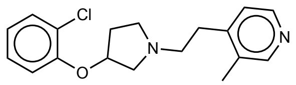	Cc1cnccc1CCN2CCC(C2)Oc3ccccc3Cl	Z2436421891<br>**2**	56 ± 2%
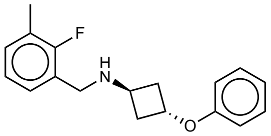	Cc1c(c(ccc1)CN[C@H]2C[C@@H](C2)Oc3ccccc3)<br>F	Z8185092308	6 ± 2%
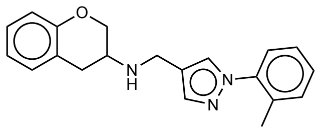	Cc1ccccc1n2cc(cn2)CNC3Cc4ccccc4OC3	Z8185092078	3 ± 1%

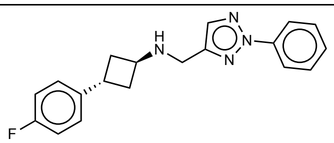	c1ccc(cc1)n2nc(cn2)CN[C@H]3C[C@@H](C3)c4cc(cc4)F	Z2213086620	27 ± 1%
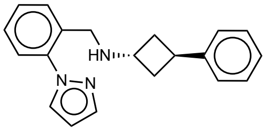	c1ccc(cc1)[C@H]2C[C@@H](C2)NCc3c(cccc3)n4nccc4	Z1973963178	1 ± 2%
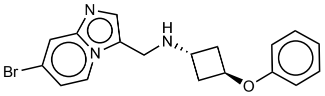	c1ccc(cc1)O[C@H]2C[C@@H](C2)NCc3n4c(nc3)cc(cc4)Br	Z8185092056	2 ± 2%
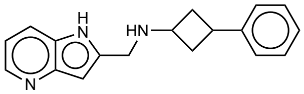	c1ccc(cc1)C2CC(C2)NCc3cc4c([nH]3)cccn4	Z3532611366	24 ± 1%
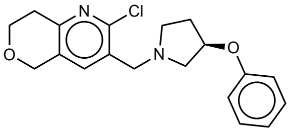	c1ccc(cc1)O[C@@H]2CCN(C2)Cc3cc4c(nc3Cl)CCOC4	Z3529190238	1 ± 1%
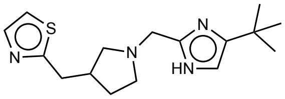	CC(C)(C)c1c[nH]c(n1)CN2CCC(C2)Cc3nccs3	Z8185092674	2 ± 3%
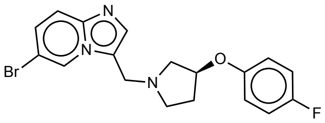	c1cc(ccc1O[C@H]2CCN(C2)Cc3cnc4n3cc(cc4)Br)F	Z8185092492	5 ± 3%
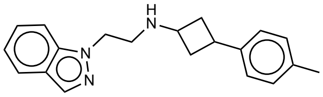	Cc1ccc(cc1)C2CC(C2)NCCn3c4ccccc4cn3	Z8185092514	23 ± 1%
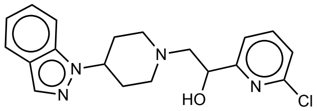	c1ccc2c(c1)cnn2C3CCN(CC3)CC(c4cccc(n4)Cl)O	Z3651347041	28 ± 1%
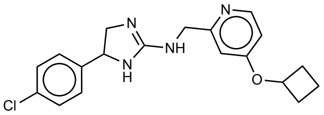	c1cc(ccc1C2CN=C(N2)NCc3cc(ccn3)OC4CCC4)Cl	Z8185092666	24 ± 1%
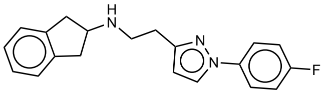	c1ccc2c(c1)CC(C2)NCCc3ccn(n3)c4ccc(cc4)F	Z1441695252<br>**1**	59 ± 1%
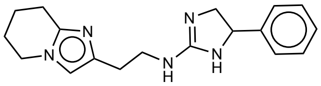	c1ccc(cc1)C2CN=C(N2)NCCc3cn4c(n3)CCCC4	Z8185092671	5 ± 2%
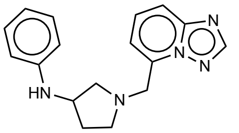	c1ccc(cc1)NC2CCN(C2)Cc3cccc4n3ncn4	Z3310096494	1 ± 2%
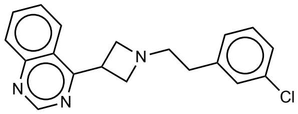	c1ccc2c(c1)c(ncn2)C3CN(C3)CCc4cccc(c4)Cl	Z3806926084	4 ± 1%

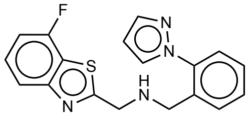	c1ccc(c(c1)CNCc2nc3cccc(c3s2)F)n4cccn4	Z8185092672	2 ± 1%
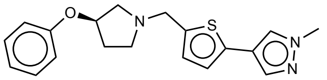	Cn1cc(cn1)c2ccc(s2)CN3CC[C@H](C3)Oc4ccccc4	Z2629646171	7 ± 1%
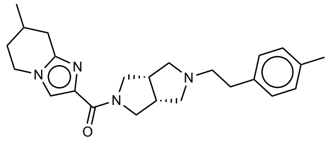	Cc1ccc(cc1)CCN2C[C@@H]3CN(C[C@@H]3C2)C(=O)c4cn5c(n4)CC(CC5)C	Z8185092530	1 ± 2%

[a] Vendor code ZXXX, manuscript compound numbers in bold. [b] Data represents mean values ± SEM of two technical replicates.

Supplementary Table 11. Chemical novelty of discovered D_2R.

#	Chemical Structure	T_c[a]	K_i (μM)[b]	ChEMBL ID	ChEMBL Structure
1	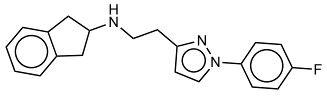	0.41	3.0 ± 0.3	CHEMBL3589575	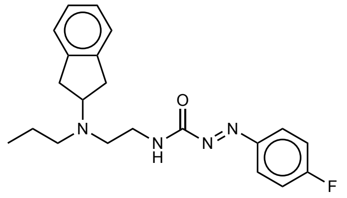
2	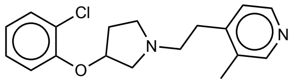	0.38	3.8 ± 0.3	CHEMBL397180	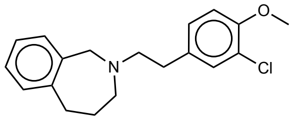

[a] Maximal Tanimoto similarity coefficient (T_c) between the compound and ChEMBL human dopamine receptor ligands with K_i < 10 μM (>11,000 compounds). Coefficients were calculated using the RDKit and Morgan2 fingerprints. [b] Data represents mean values ± SEM from three independent experiments.

Supplementary Table 12. Chemical structures and $A_{2A}R$ radioligand displacement data.

Chemical Structure	SMILES	Vendor code[a]	Displacement at 20 μM (%)[b]
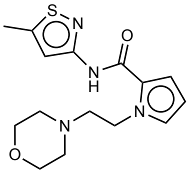	Cc1cc(ns1)NC(=O)c2cccn2CCN3CCOCC3	Z3591989733	19±1%
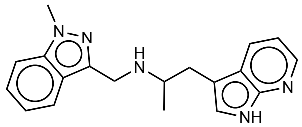	CC(Cc1c[nH]c2c1cccn2)NCc3c4ccccc4n(n3)C	Z7272600070	20±1%
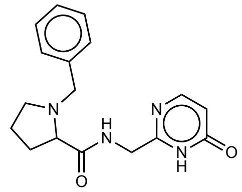	c1ccc(cc1)CN2CCCC2C(=O)NCc3[nH]c(=O)ccn3	Z4219981720	6±4%
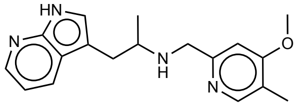	Cc1cnc(cc1OC)CNC(C)Cc2c[nH]c3c2cccn3	Z7326877935	13±2%
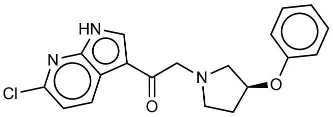	c1ccc(cc1)O[C@H]2CCN(C2)CC(=O)c3c[nH]c4c3ccc(n4)Cl	Z8854579348 **4**	1.4 μM
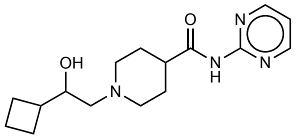	c1cnc(nc1)NC(=O)C2CCN(CC2)CC(C3CCC3)O	Z3292775568	1±2%
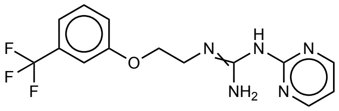	c1cc(cc(c1)OCCN=C(N)Nc2ncccn2)C(F)(F)F	Z8854579346	23±1%
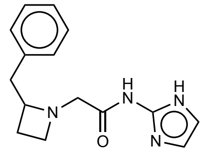	c1ccc(cc1)CC2CCN2CC(=O)Nc3[nH]ccn3	Z6743522026	5±3%
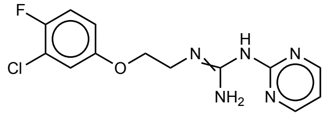	c1cnc(nc1)NC(=NCCOc2ccc(c(c2)Cl)F)N	Z8854579360	26±3%
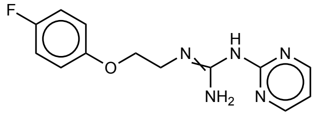	c1cnc(nc1)NC(=NCCOc2ccc(cc2)F)N	Z8854579344	17±1%
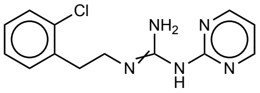	c1ccc(c(c1)CCN=C(N)Nc2ncccn2)Cl	Z8854579337	29±4%

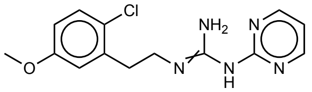	COc1ccc(c(c1)CCN=C(N)Nc2ncccn2)Cl	Z8854579336	39±1%
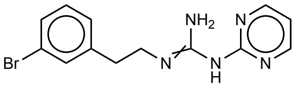	c1cc(cc(c1)Br)CCN=C(N)Nc2ncccn2	Z8854579355	28±1%
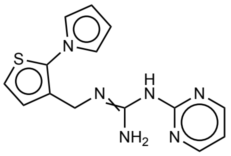	c1ccn(c1)c2c(ccs2)CN=C(N)Nc3ncccn3	Z8854579347	32±4%
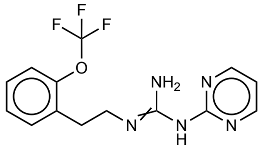	c1ccc(c(c1)CCN=C(N)Nc2ncccn2)OC(F)(F)F	Z8854579353	22±5%
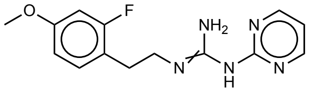	COc1ccc(c(c1)F)CCN=C(N)Nc2ncccn2	Z8854579362	12±4%
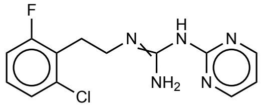	c1cc(c(c(c1)Cl)CCN=C(N)Nc2ncccn2)F	Z8854579358	16±4%
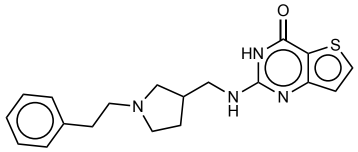	c1ccc(cc1)CCN2CCC(C2)CNc3[nH]c(=O)c4c(n3)ccs4	Z5332306614	8±1%
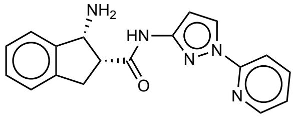	c1ccc2c(c1)C[C@H]([C@H]2N)C(=O)Nc3ccn(n3)c4ccccn4	Z8854579342	30±2%
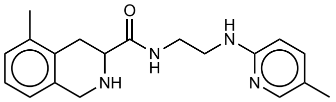	Cc1ccc(nc1)NCCNC(=O)C2Cc3c(cccc3CN2)C	Z8854579335	6±2%
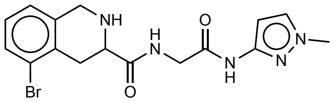	Cn1ccc(n1)NC(=O)CNC(=O)C2Cc3c(cccc3Br)CN2	Z8854579367	10±2%
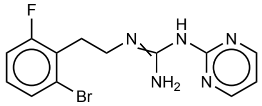	c1cc(c(c(c1)Br)CCN=C(N)Nc2ncccn2)F	Z8854579363	20±1%
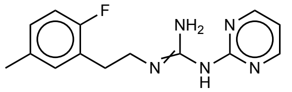	Cc1ccc(c(c1)CCN=C(N)Nc2ncccn2)F	Z8854579366	15±1%
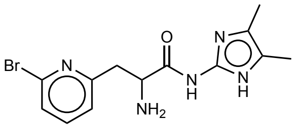	Cc1c(nc([nH]1)NC(=O)C(Cc2cccc(n2)Br)N)C	Z6382061841	2±2%
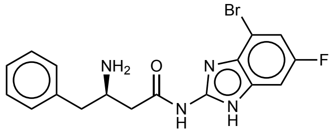	c1ccc(cc1)C[C@H](CC(=O)Nc2[nH]c3cc(cc(c3n2)Br)F)N	Z8854579338	49±2%

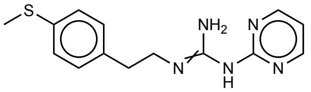	CSc1ccc(cc1)CCN=C(N)Nc2ncccn2	Z8854579356	15±1%
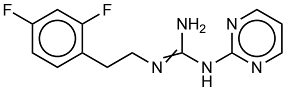	c1cnc(nc1)NC(=NCCc2ccc(cc2F)F)N	Z8854579368	14±1%
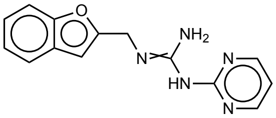	c1ccc2c(c1)cc(o2)CN=C(N)Nc3ncccn3	Z8854579345	32±2%
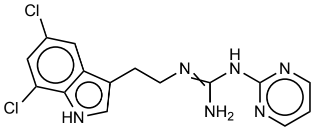	c1cnc(nc1)NC(=NCCc2c[nH]c3c2cc(cc3Cl)Cl)N	Z8854579357 **5**	20±3.0 μM
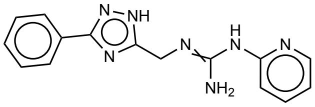	c1ccc(cc1)c2nc([nH]n2)CN=C(N)Nc3ccccn3	Z2518713795	23±3%
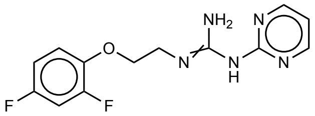	c1cnc(nc1)NC(=NCCOc2ccc(cc2F)F)N	Z8854579369	2±3%
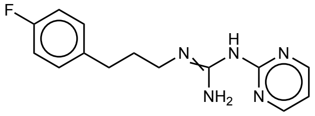	c1cnc(nc1)NC(=NCCCc2ccc(cc2)F)N	Z8854579364	17±4%
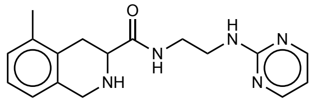	Cc1cccc2c1CC(NC2)C(=O)NCCNc3ncccn3	Z8854579354	1±1%
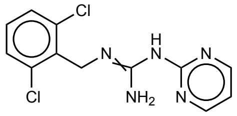	c1cc(c(c(c1)Cl)CN=C(N)Nc2ncccn2)Cl	Z8861112994	32±2%
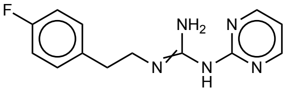	c1cnc(nc1)NC(=NCCc2ccc(cc2)F)N	Z8854579370	11±1%
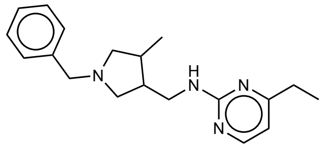	CCc1ccnc(n1)NCC2CN(CC2C)Cc3ccccc3	Z5471612810	2±1%
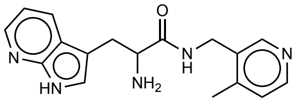	Cc1ccncc1CNC(=O)C(Cc2c[nH]c3c2cccn3)N	Z8857701713	3±2%
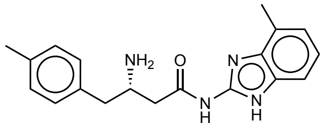	Cc1ccc(cc1)C[C@@H](CC(=O)Nc2[nH]c3cccc(c3n 2)C)N	Z8854579350	30±3%
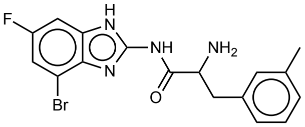	Cc1cccc(c1)CC(C(=O)Nc2[nH]c3cc(cc(c3n2)Br)F) N	Z8854579339 **3**	2.5 μM

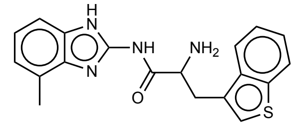	Cc1cccc2c1nc([nH]2)NC(=O)C(Cc3csc4c3cccc4)N	Z8854579351	31±1%
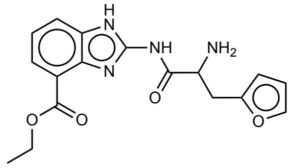	CCOC(=O)c1cccc2c1nc([nH]2)NC(=O)C(Cc3ccco3)N	Z8857701715 **6**	1.3 μM
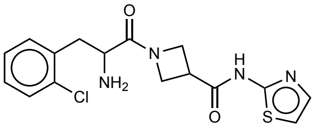	c1ccc(c(c1)CC(C(=O)N2CC(C2)C(=O)Nc3nccs3)N)Cl	Z6437654059	3±2%
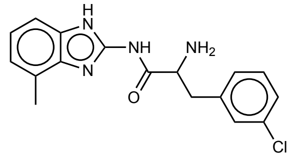	Cc1cccc2c1nc([nH]2)NC(=O)C(Cc3cccc(c3)Cl)N	Z8854579352	57±4%
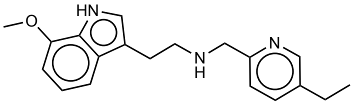	CCc1ccc(nc1)CNCCc2c[nH]c3c2cccc3OC	Z3765568162	16±4%
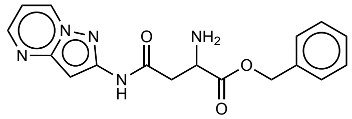	c1ccc(cc1)COC(=O)C(CC(=O)Nc2cc3ncccn3n2)N	Z8854579341	2±2%

[a] Vendor code ZXXX, manuscript compound numbers in bold. [b] Percentage displacement data represents mean values ± SEM of two technical replicates. K_i values obtained from fitting to concentration-response curve from two technical replicates, except for compound **5** (mean ± SEM) which was tested in three independent experiments.

Supplementary Table 13. $A_{2A}R$ ligands and the most similar known adenosine receptor ligand.

#	Chemical Structure	T_c[a]	Activity[b]	ChEMBL ID	ChEMBL Structure
3	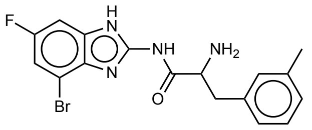	0.38	2.5 μM	CHEMBL3763215	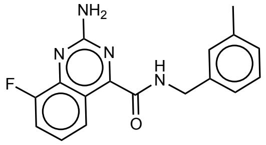
4	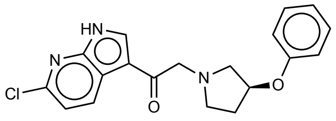	0.36	1.4 μM	CHEMBL471853	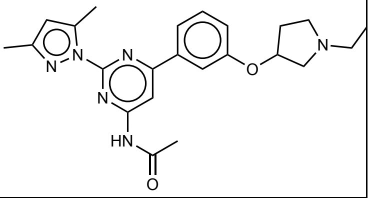
5	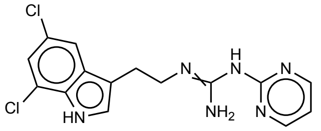	0.37	20±3.0 μM	CHEMBL1098444	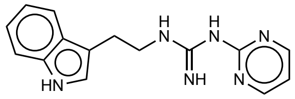
6	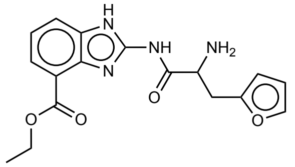	0.37	1.3 μM	CHEMBL3091695	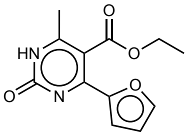

[a] Maximal Tanimoto similarity coefficient (T_c) between the compound and ChEMBL human adenosine receptor ligands with K_i < 10 μM (>10,000 compounds). Coefficients were calculated using the RDKit and Morgan2 fingerprints. [b] K_i values obtained from fitting to concentration-response curve from two technical replicates, except for compound **5** (mean ±SEM) which was tested in three independent experiments.

Supplementary Table 14. Dual-target ligand and the most similar known dopamine and adenosine receptor ligands.

#	Chemical Structure	Target	T_c[a]	K_i (μM)[b]	ChEMBL ID	ChEMBL Structure
5	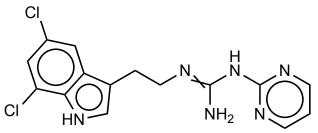	$A_{2A}R$	0.37	20±3.0	CHEMBL1098444	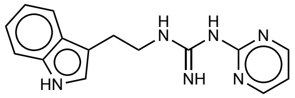
		D_2R	0.28	14±0.7	CHEMBL267014	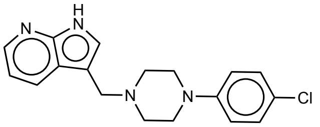

[a] Maximal Tanimoto similarity coefficient (T_c) between compound **5** and ChEMBL human adenosine and dopamine receptor ligands with K_i < 10 μM (>21,000 compounds). Coefficients were calculated using the RDKit and Morgan2 fingerprints. [b] Data represents mean values ± SEM from three independent experiments.

Supplementary Figures

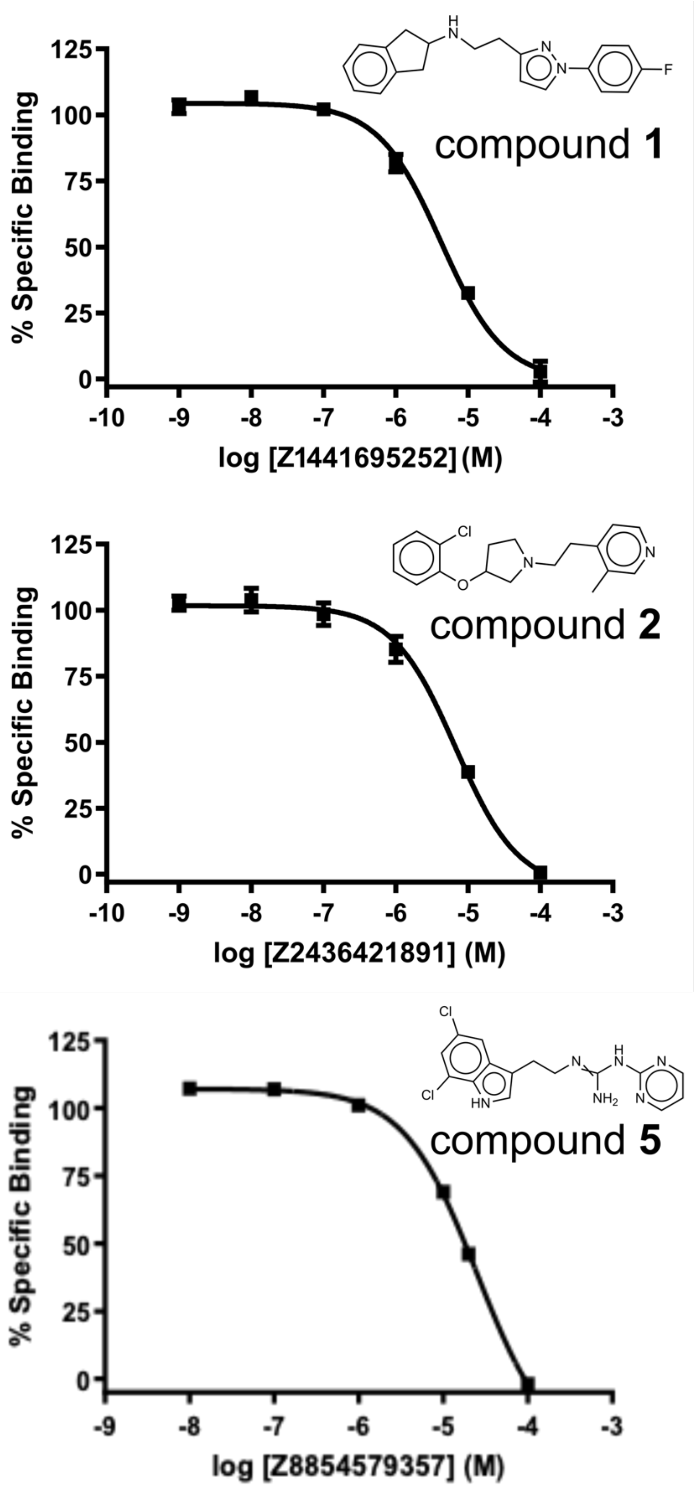

Supplementary Figure 15. Radioligand displacement binding curves of discovered D_2R ligands. Percentage D_2R radioligand displacement by compounds **1, 2**, and **5** in function of their concentration. Data points represent mean ± SEM from three independent experiments.

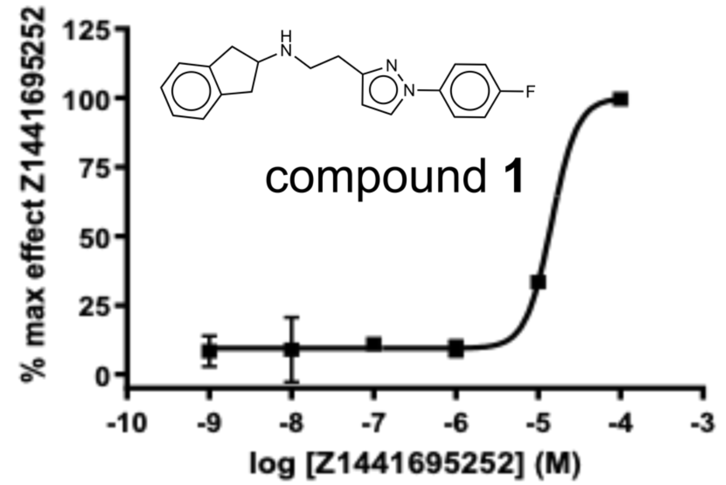

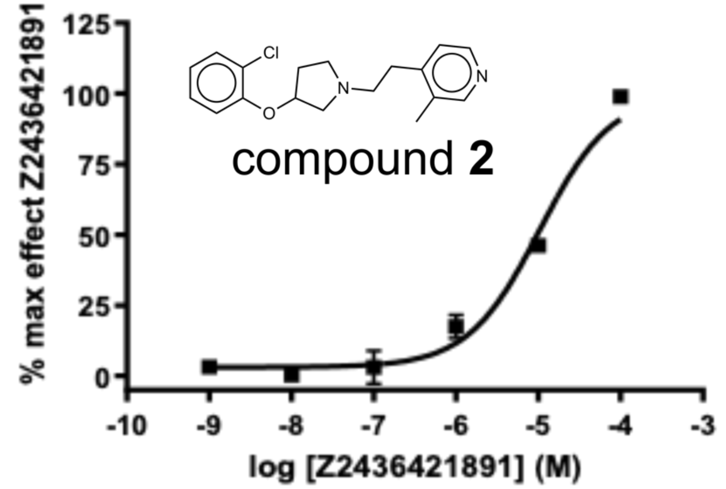

Supplementary Figure 16. Functional assay curves of discovered D_2R ligands. Representative concentration-response curves of compounds **1** and **2** in functional assays at the D_2R. Data points represent individual measurements from a single experiment and the corresponding error bars represent the error of the curve fit on those data points.

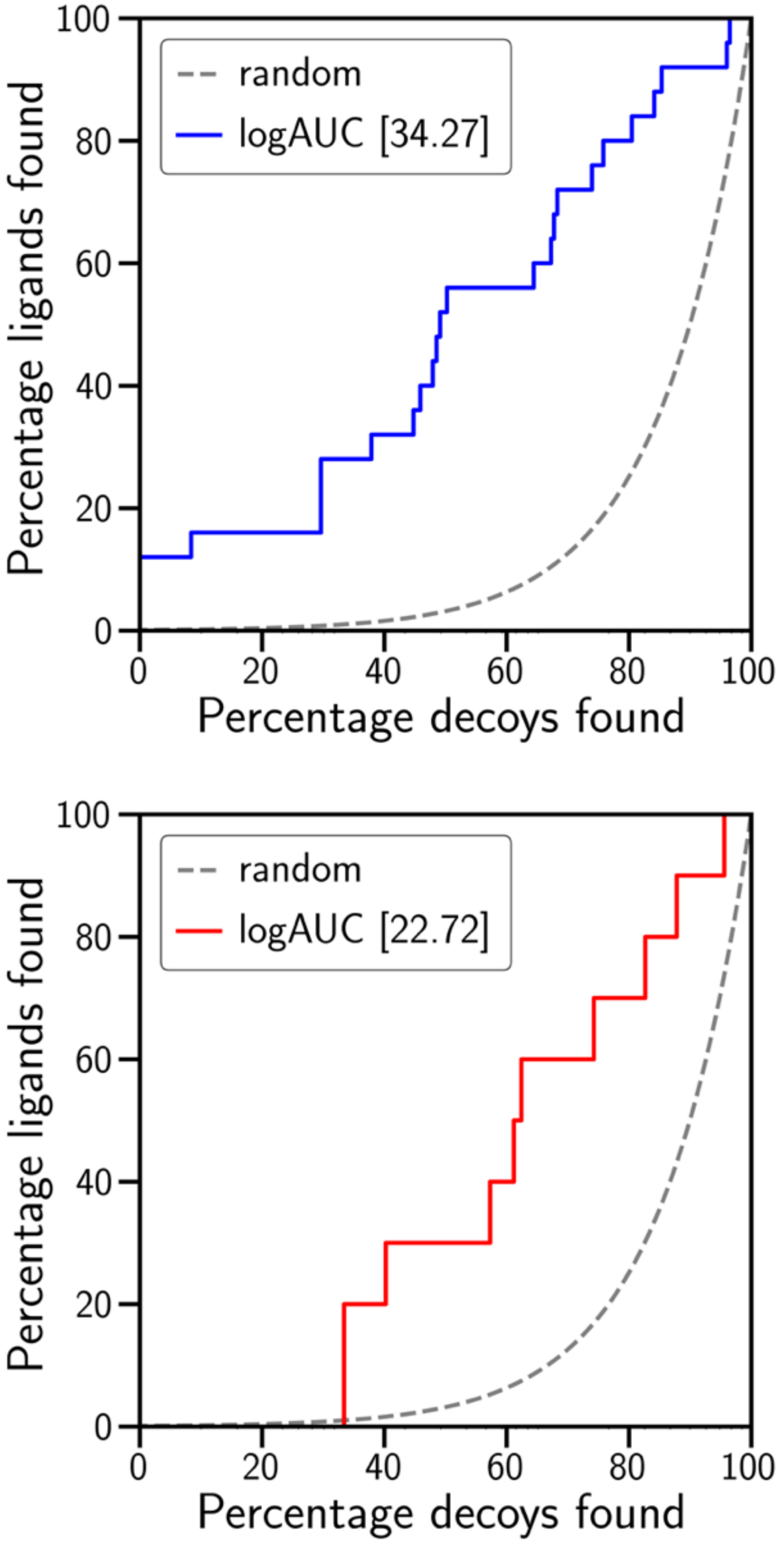

Supplementary Figure 17. Ligand enrichment curves for $A_{2A}R$ and D_2R models. Logarithmic receiver operator characteristic (ROC) curves describing the enrichment of known binders of the (a) $A_{2A}R$ and (b) D_2R over corresponding property-matched decoys.

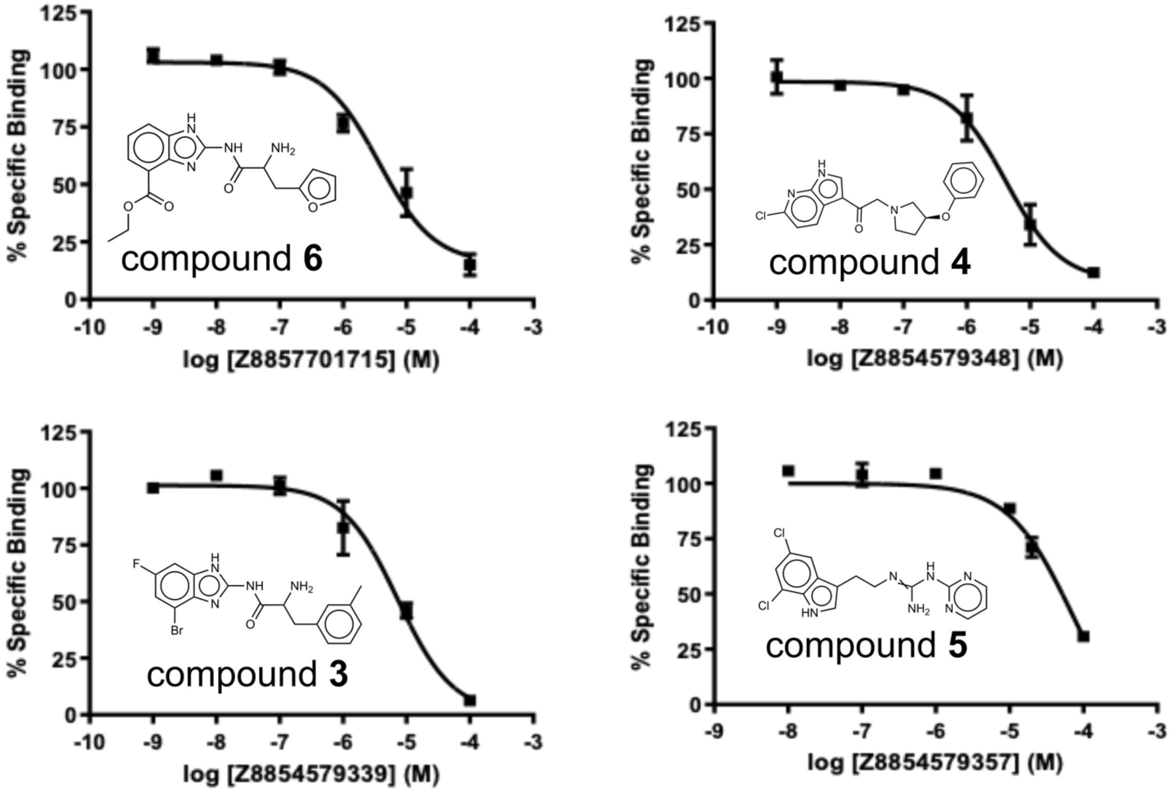

Supplementary Figure 18. Radioligand displacement binding curves of discovered A_{2A}R ligands. A_{2A}R radioligand displacement by compounds **3-6** in function of their concentration. Data points represent mean ± SEM from two technical replicates for compound **3, 4** and **6**, and three independent experiments for compound **5**.

LC-MS Spectral Data

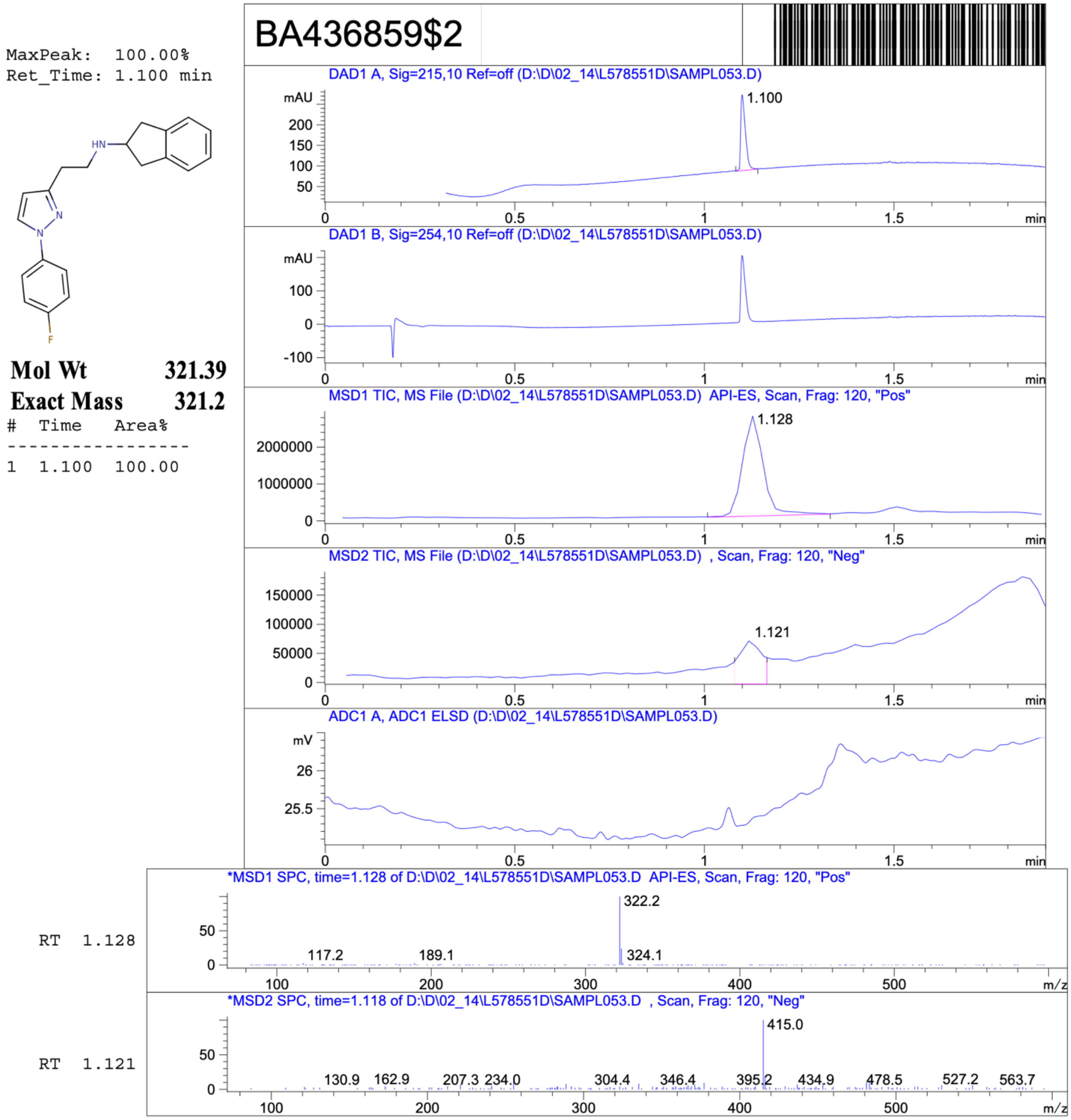

Supplementary Figure 19. LC-MS data for compound 1. Chemical characterization of compound **1** (Z1441695252) by chromatography (top) and mass-spectrometry (bottom).

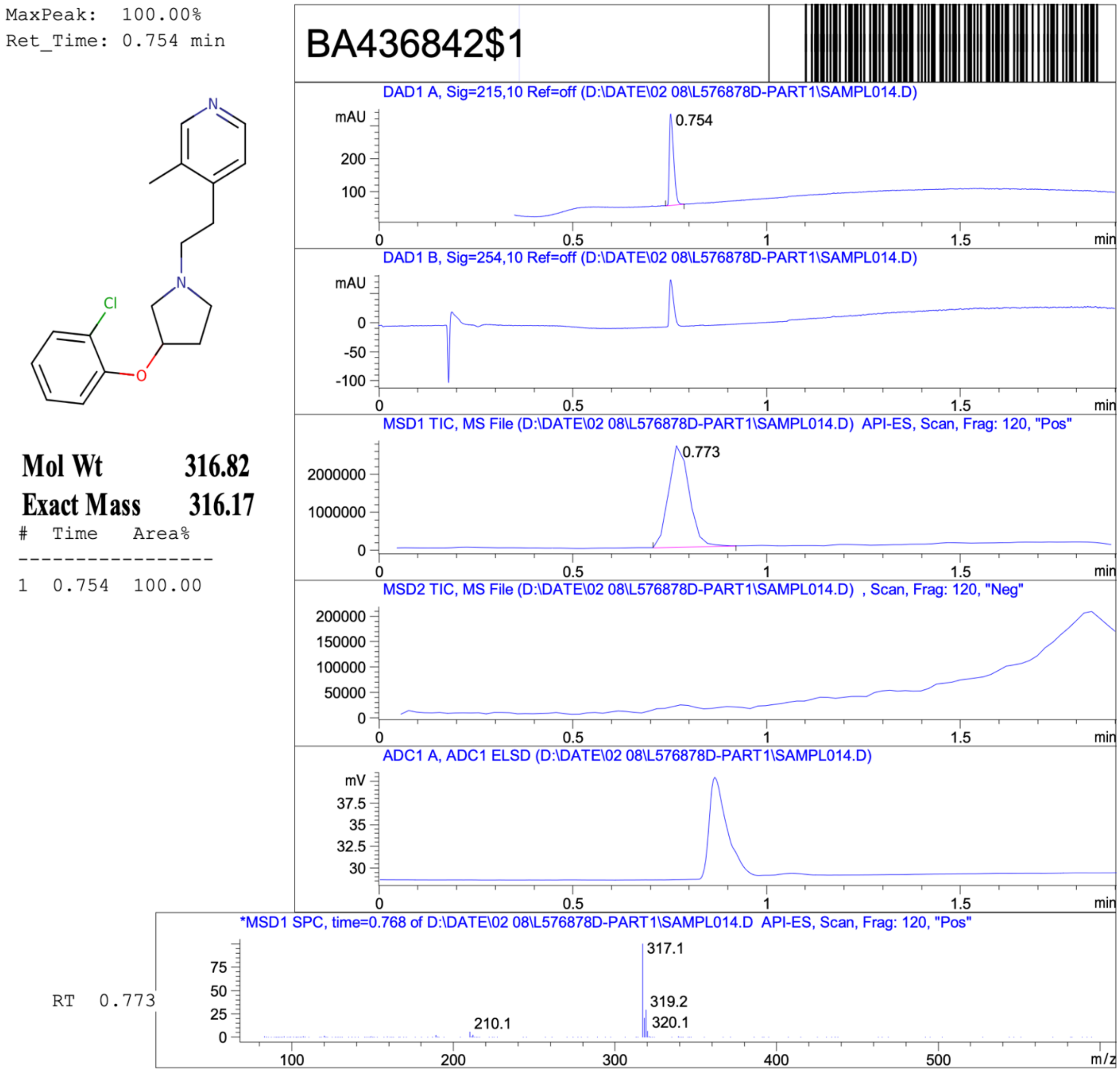

Supplementary Figure 20. LC-MS data for compound 2. Chemical characterization of compound **2** (Z2436421891) by chromatography (top) and mass-spectrometry (bottom).

MaxPeak: 96.63%
Ret_Time: 0.881 min

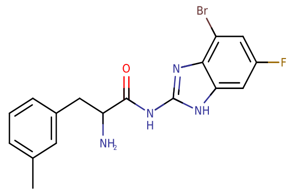

Mol Wt 391.24
Exact Mass 390.07

#	Time	Area%
1	0.603	3.37
2	0.881	96.63

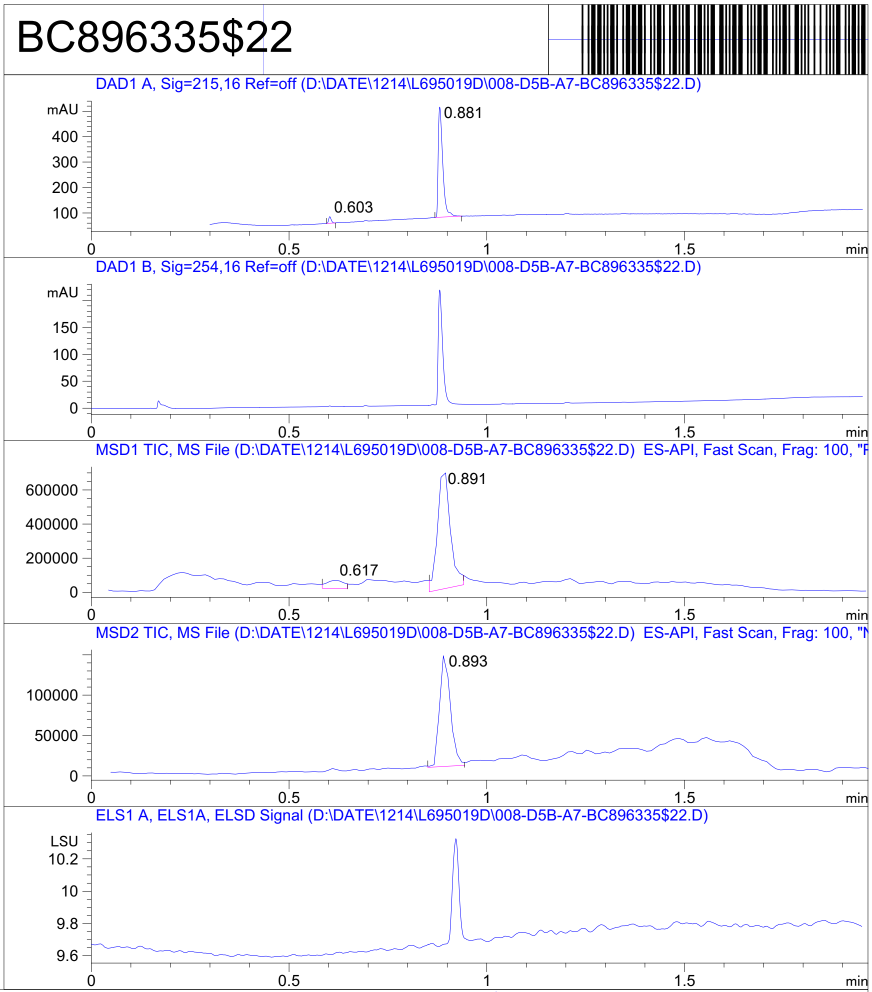

RT 0.617

RT 0.891

RT 0.893

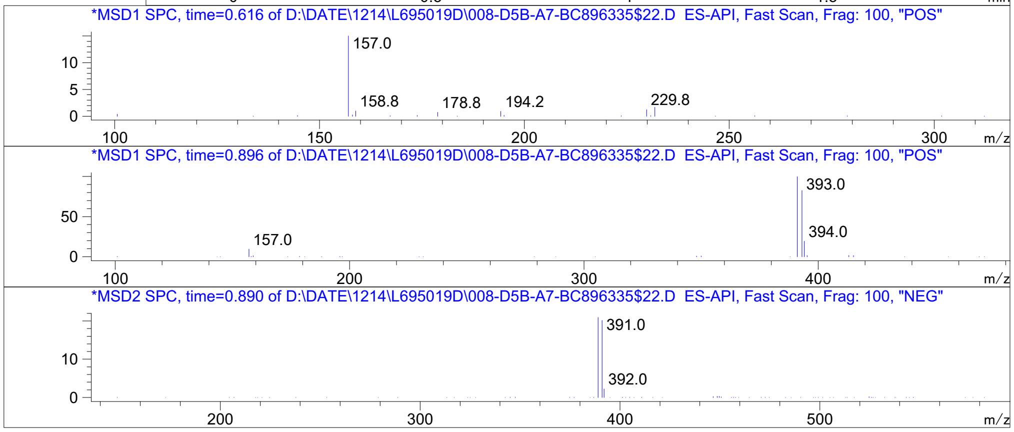

Inj.Date 12/14/2023 E Acq. Method C:\Users\ -> ->

Supplementary Figure 21. LC-MS data for compound 3. Chemical characterization of compound **3** (Z8854579339) by chromatography (top) and mass-spectrometry (bottom).

MaxPeak: 97.25%
Ret_Time: 0.869 min

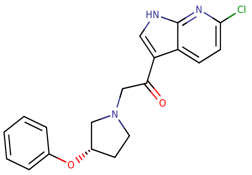

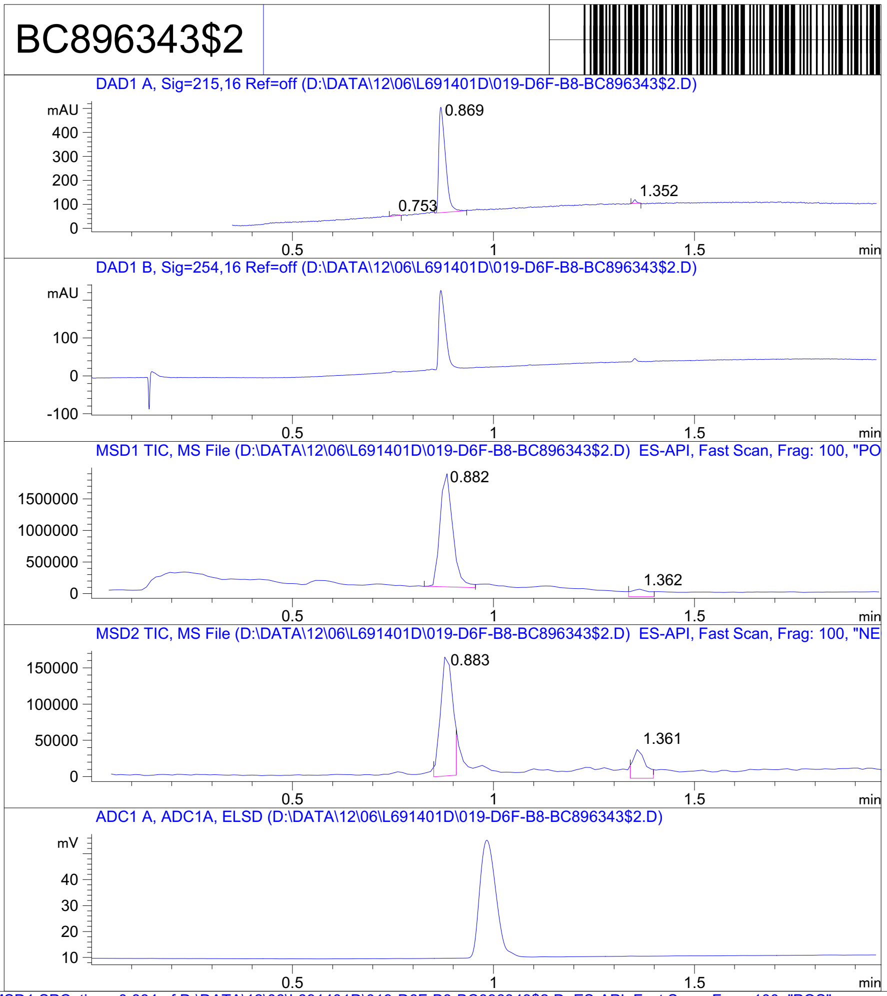

Mol Wt 355.82
Exact Mass 355.13

#	Time	Area%
1	0.753	1.09
2	0.869	97.25
3	1.352	1.65

RT 0.882

RT 1.362

RT 0.883

RT 1.361

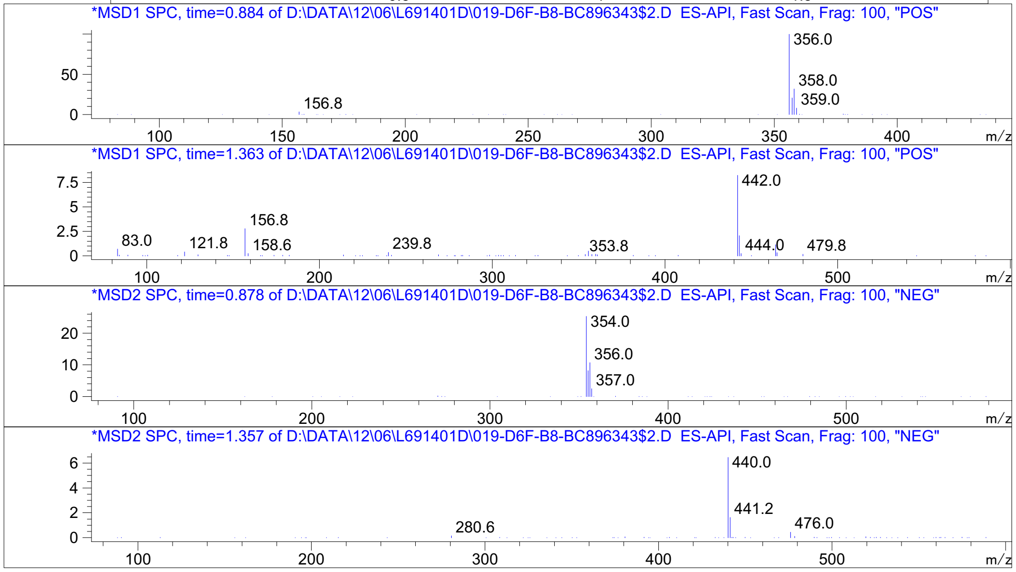

Inj.Date 12/6/2023 CH <invalid> -3- Acq. Method C:\Users\ -> ->

Supplementary Figure 22. LC-MS data for compound 4. Chemical characterization of compound **4** (Z8854579348) by chromatography (top) and mass-spectrometry (bottom).

MaxPeak: 100.00%
Ret_Time: 0.744 min

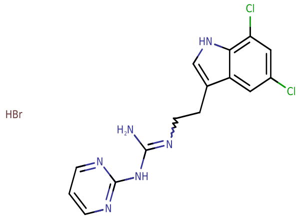

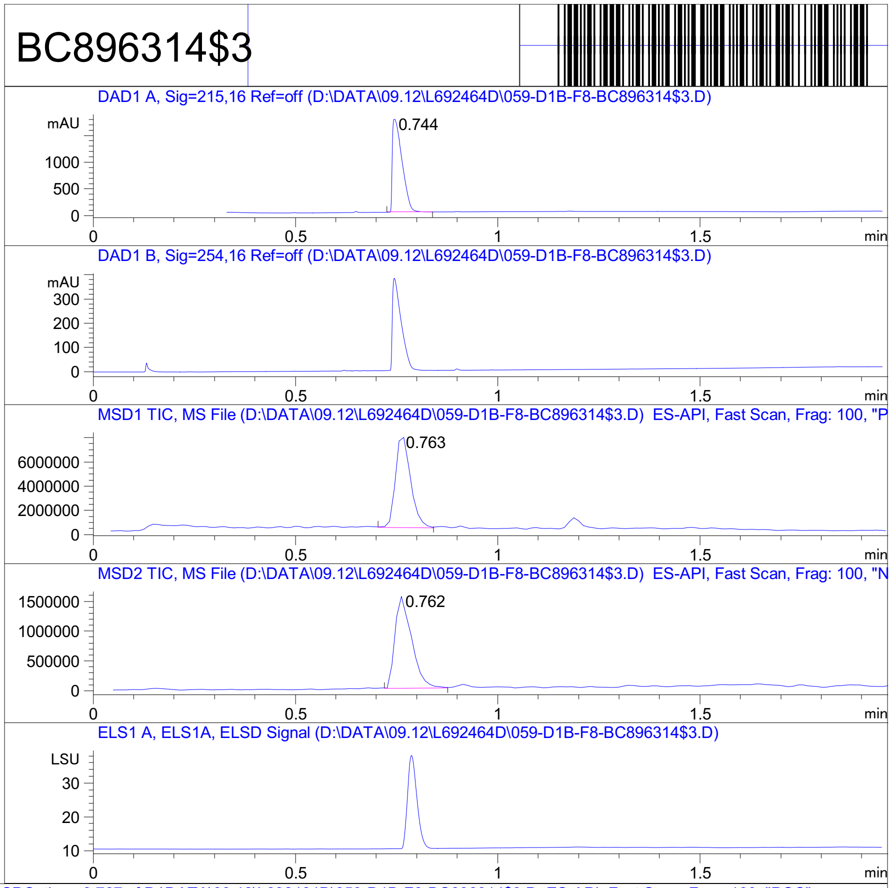

Mol Wt 430.13

Exact Mass 348.08

#	Time	Area%
1	0.744	100.00

RT 0.763

RT 0.762

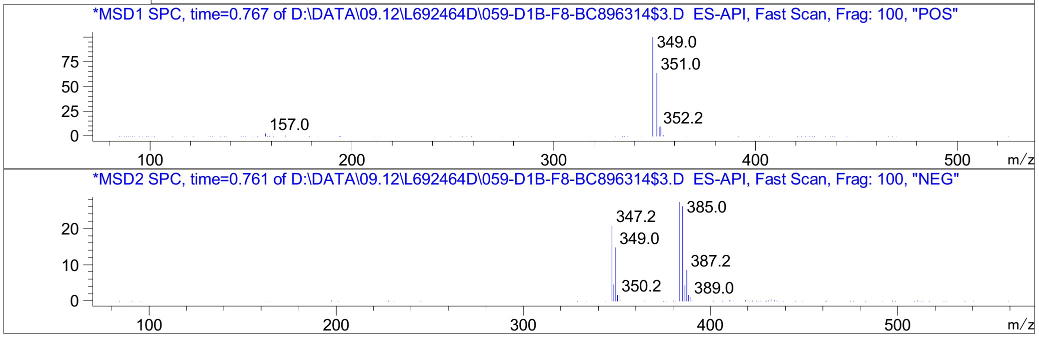

Inj.Date 12/8/2023 M 33

Supplementary Figure 23. LC-MS data for compound 5. Chemical characterization of compound **5** (Z8854579357) by chromatography (top) and mass-spectrometry (bottom).

MaxPeak: 96.19%
Ret_Time: 0.907 min

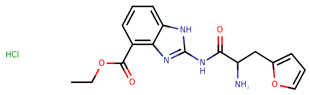

Mol Wt 378.81
Exact Mass 342.14

#	Time	Area%
1	0.769	3.81
2	0.907	96.19

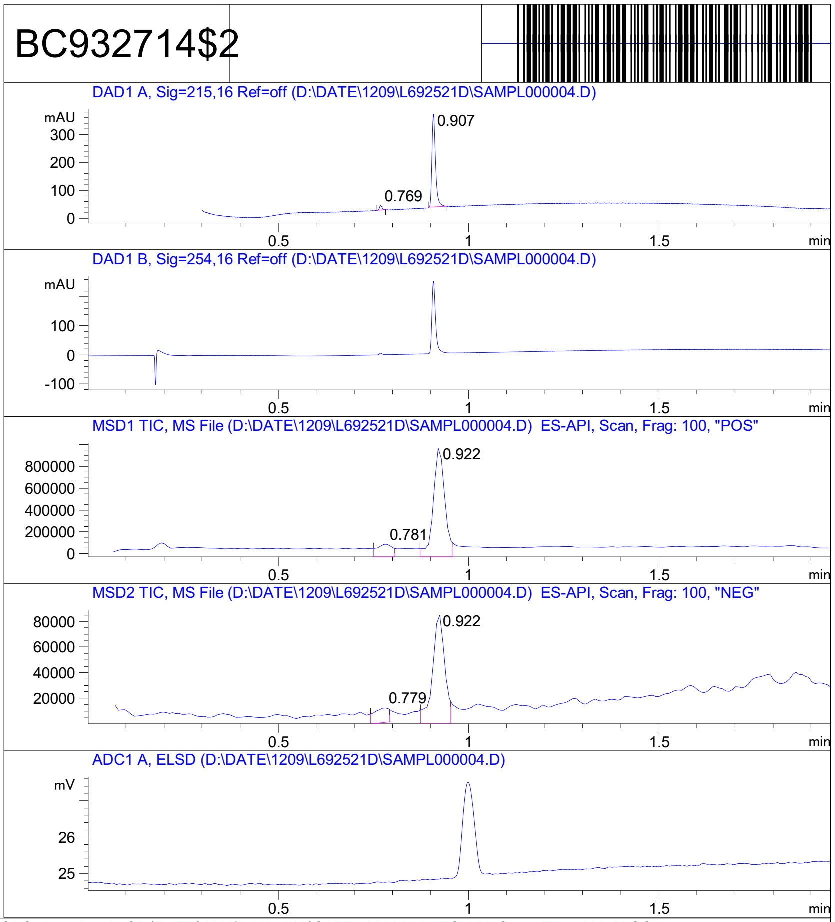

RT 0.781

RT 0.922

RT 0.779

RT 0.922

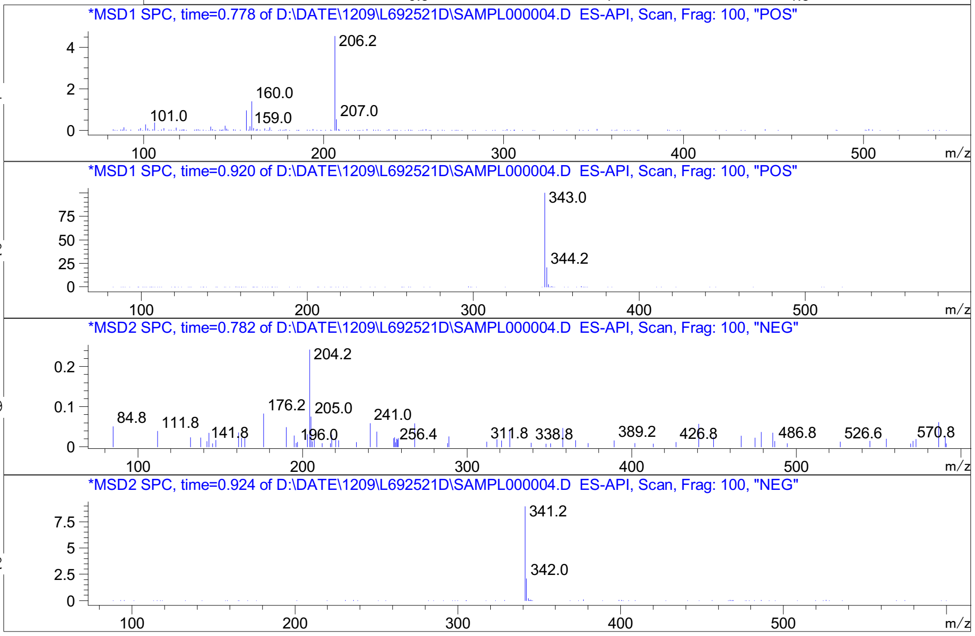

Inj.Date 12/8/2023 CH -6- Acq. Method C:\CHEM32\-> ->

Supplementary Figure 24. LC-MS data for compound 6. Chemical characterization of compound **6** (Z8857701715) by chromatography (top) and mass-spectrometry (bottom).

NMR Spectra

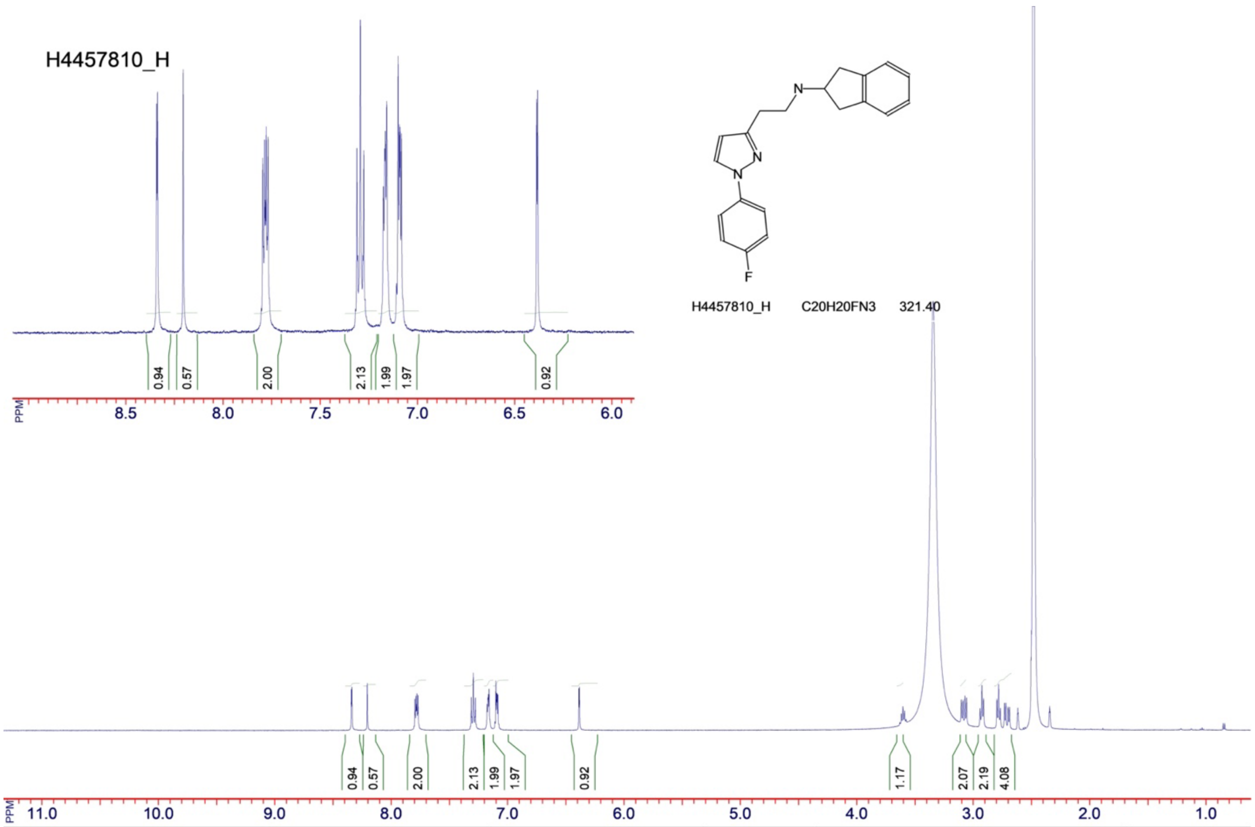

File name: H4457810_H	Operator: Victorova	SF: 499.8206 MHz	NSC: 0	PW: 4.20 usec, RG: 42	SI: 131072
Date: 04-Sep-2023	Solvent: dmso	SW: 9921 Hz	TE: 302 K	AQ: 3.23 sec, RD: 0.00 sec	

Supplementary Figure 25. ^{1}H-NMR data for compound 1. Chemical characterization of compound **1** (Z1441695252) by proton nuclear magnetic resonance.

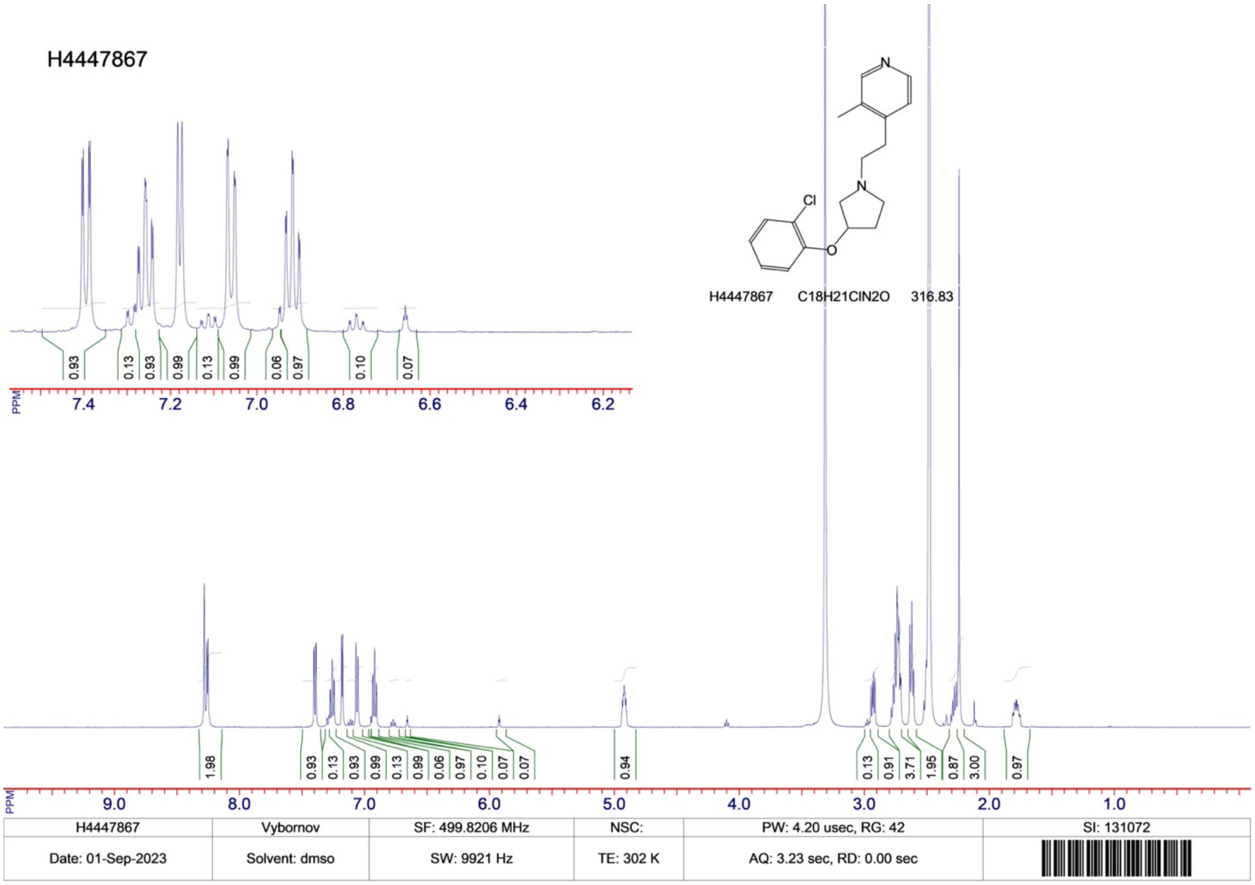

Supplementary Figure 26. ^{1}H-NMR data for compound 2. Chemical characterization of compound **2** (Z2436421891) by proton nuclear magnetic resonance.

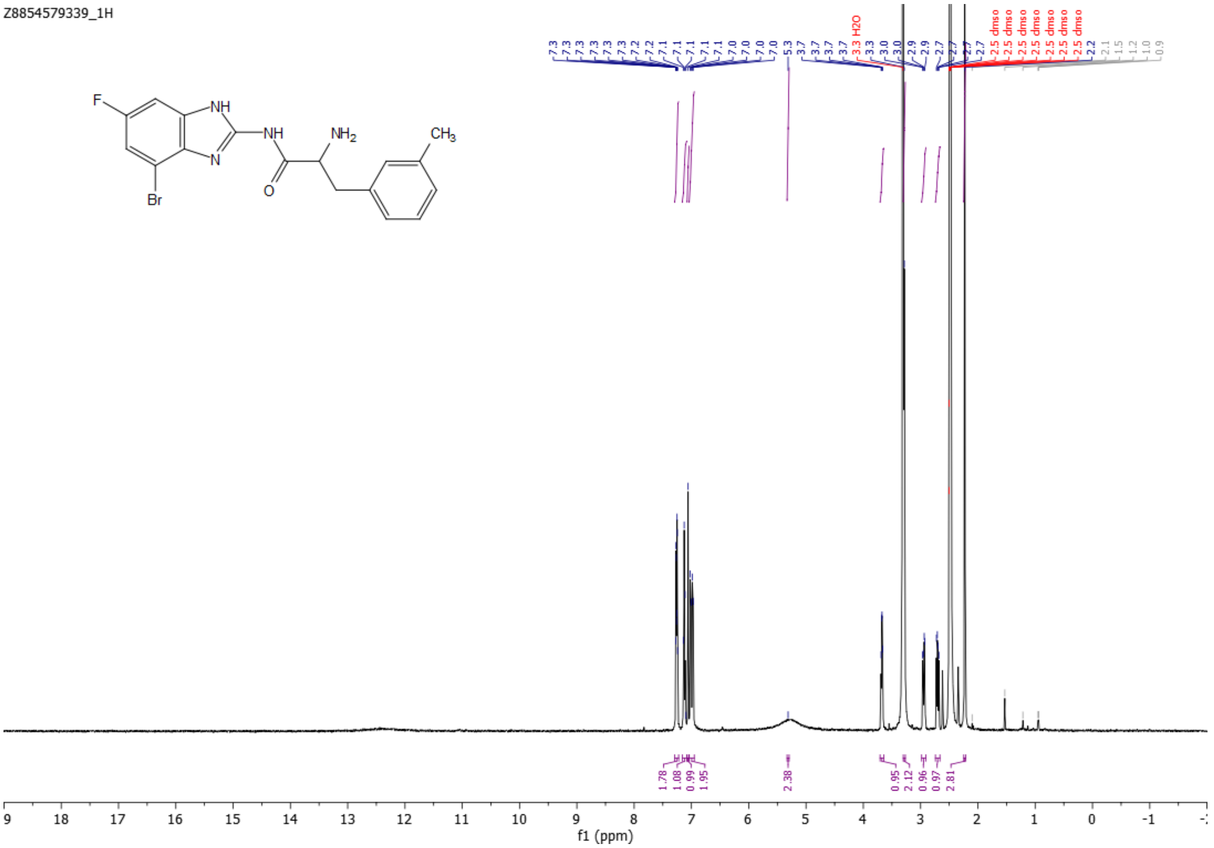

Supplementary Figure 27. ^{1}H-NMR data for compound 3. Chemical characterization of compound **3** (Z8854579339) by proton nuclear magnetic resonance.

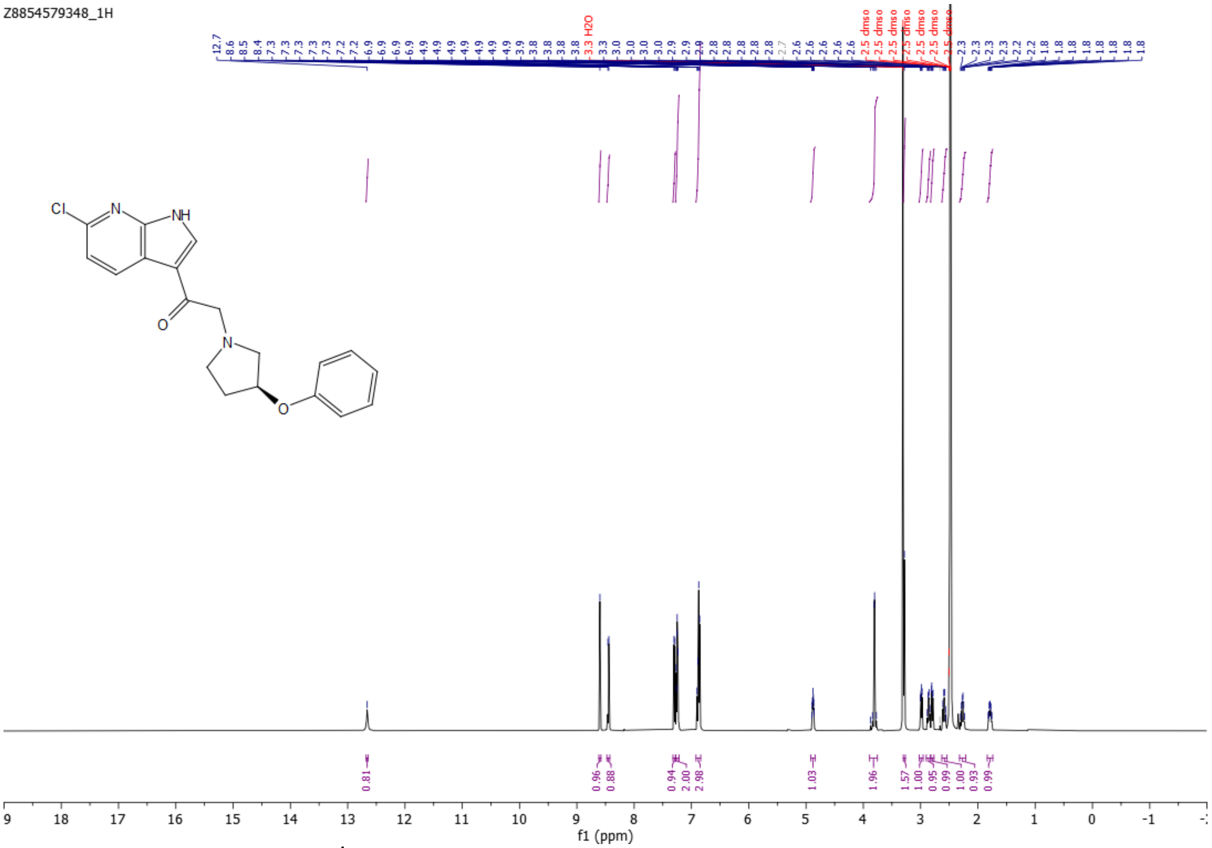

Supplementary Figure 28. ^{1}H-NMR data for compound 4. Chemical characterization of compound **4** (Z8854579348) by proton nuclear magnetic resonance.

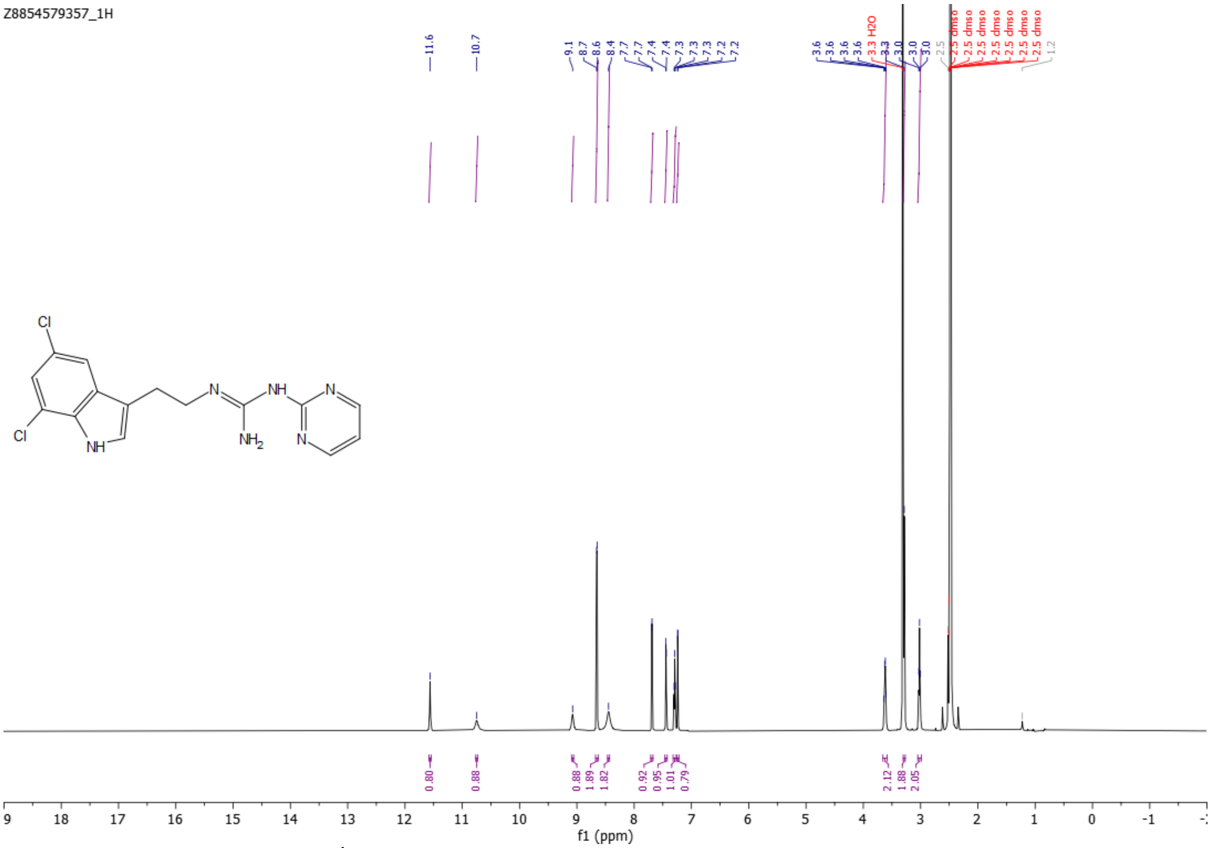

Supplementary Figure 29. ^{1}H-NMR data for compound 5. Chemical characterization of compound **5** (Z8854579357) by proton nuclear magnetic resonance.

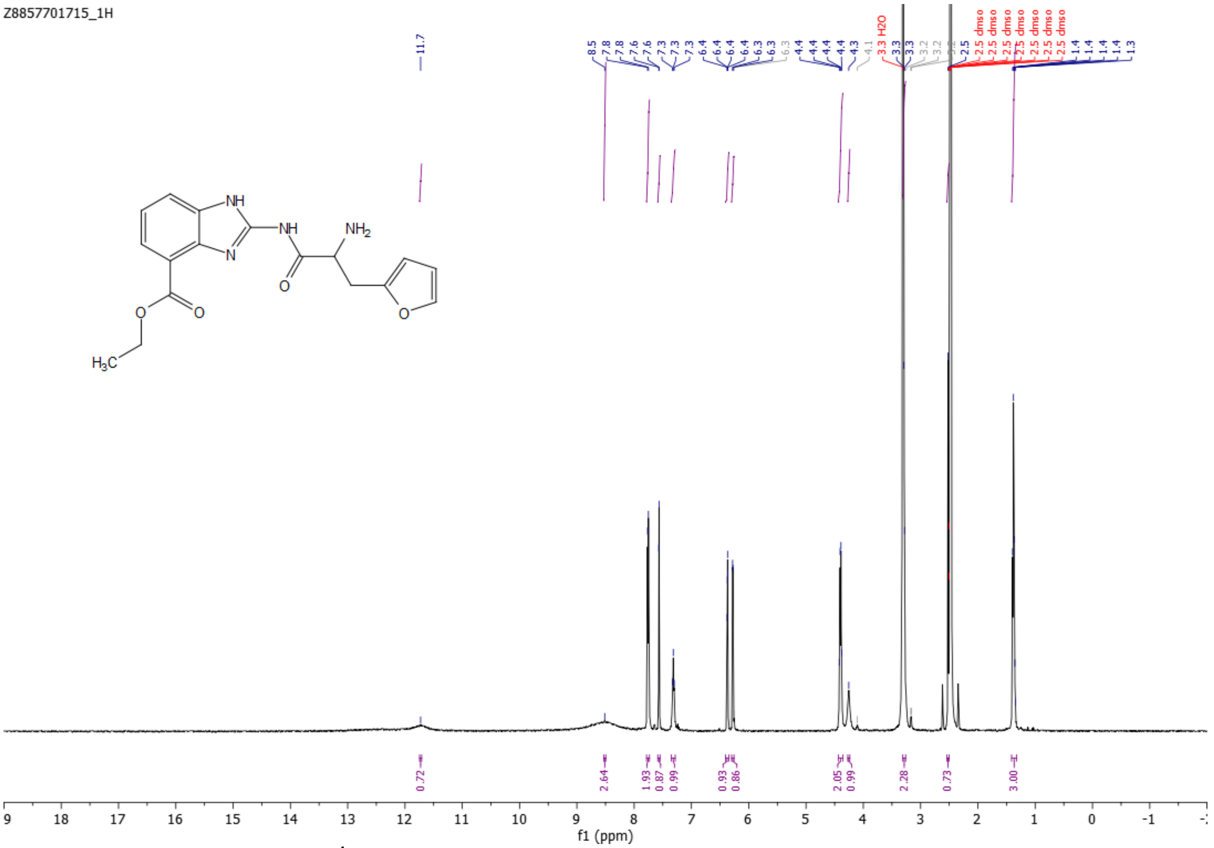

Supplementary Figure 30. ^{1}H-NMR data for compound 6. Chemical characterization of compound **6** (Z8857701715) by proton nuclear magnetic resonance.

Supplementary References

1. Liu W., *et al*. Structural basis for allosteric regulation of GPCRs by sodium ions. *Science*, **337**, 232-236 (2012).
2. Lyu J., *et al.* Ultra-large library docking for discovering new chemotypes. *Nature*, **566**, 224-229 (2019).
3. Beatty J. W., *et al.* Discovery of Potent and Selective Non-Nucleotide Small Molecule Inhibitors of CD73. *J. Med. Chem.*, **63**, 3935-3955 (2020).
4. Wang S., *et al*. Structure of the D2 dopamine receptor bound to the atypical antipsychotic drug risperidone. *Nature*, **555**, 269-273 (2018).
5. Davies T. G., *et al*. Monoacidic Inhibitors of the Kelch-like ECH-Associated Protein 1: Nuclear Factor Erythroid 2-Related Factor 2 (KEAP1:NRF2) Protein-Protein Interaction with High Cell Potency Identified by Fragment-Based Discovery. *J. Med. Chem.*, **59**, 3991-4006 (2016).
6. Mesecar A. D., 2020, https://www.rcsb.org/structure/6W63
7. Visnes T., *et al*. Small-molecule inhibitor of OGG1 suppresses proinflammatory gene expression and inflammation. *Science*, **362**, 834-839 (2018).
8. Stachel S. J., *et al*. Identification of potent inhibitors of the sortilin-progranulin interaction. *Bioorg. Med. Chem. Lett.*, **30**, 127403 (2020).
9. Ohno Y., *et al*. In Vitro Pharmacological Profile of KW-6356, a Novel Adenosine A_{2A} Receptor Antagonist/Inverse Agonist. *Mol. Pharmacol.*, **103**, 6, 311-324 (2023).
10. Xu P., *et al*. Structures of the human dopamine D3 receptor-G_i complexes. *Mol. Cell,* **81**, 6, 1147-1159.e4 (2021).
11. Alvarsson, J. *et al*. Predicting With Confidence: Using Conformal Prediction in Drug Discovery. *J. Pharm. Sci.*, **110**, 42-49 (2021).
12. Tamma, P. D. *et al*. A Primer on AmpC β-Lactamases: Necessary Knowledge for an Increasingly Multidrug-resistant World. *Clin. Infect. Dis.*, **69**, 1446-1455 (2019).
13. Ullrich, S., & Nitsche C. The SARS-CoV-2 main protease as drug target. *Med. Chem. Lett.*, **30**, 127377 (2020).
14. Bellmann L., Penner P., Gastreich M. & Rarey M. Comparison of Combinatorial Fragment Spaces and Its Application to Ultralarge Make-on-Demand Compound Catalogs. *J. Chem. Inf. Model*. **62**, 553-566 (2022).
