# Supplementary material for: Rapid traversal of vast chemical space using machine learning-guided docking screens
Source: Nat Comput Sci. 2025 Mar 13;5(4):301–12. doi: 10.1038/s43588-025-00777-x (PMC12021657; doi:10.1038/s43588-025-00777-x)
Supplement: Supplementary file 2 — Reporting Summary [file 43588_2025_777_MOESM2_ESM.pdf]

nature portfolio

Corresponding author(s): Andreas Luttens, Maria Isabel Loza, Ulf Norinder, Jens Carlsson

Last updated by author(s): January 20th, 2025

Reporting Summary

Nature Portfolio wishes to improve the reproducibility of the work that we publish. This form provides structure for consistency and transparency in reporting. For further information on Nature Portfolio policies, see our Editorial Policies and the Editorial Policy Checklist.

Statistics

For all statistical analyses, confirm that the following items are present in the figure legend, table legend, main text, or Methods section.

n/a	Confirmed	
☐	☒	The exact sample size (*n*) for each experimental group/condition, given as a discrete number and unit of measurement
☐	☒	A statement on whether measurements were taken from distinct samples or whether the same sample was measured repeatedly
☐	☒	The statistical test(s) used AND whether they are one- or two-sided<br>*Only common tests should be described solely by name; describe more complex techniques in the Methods section.*
☒	☐	A description of all covariates tested
☒	☐	A description of any assumptions or corrections, such as tests of normality and adjustment for multiple comparisons
☐	☒	A full description of the statistical parameters including central tendency (e.g. means) or other basic estimates (e.g. regression coefficient) AND variation (e.g. standard deviation) or associated estimates of uncertainty (e.g. confidence intervals)
☐	☒	For null hypothesis testing, the test statistic (e.g. *F*, *t*, *r*) with confidence intervals, effect sizes, degrees of freedom and *P* value noted<br>*Give P values as exact values whenever suitable.*
☒	☐	For Bayesian analysis, information on the choice of priors and Markov chain Monte Carlo settings
☒	☐	For hierarchical and complex designs, identification of the appropriate level for tests and full reporting of outcomes
☒	☐	Estimates of effect sizes (e.g. Cohen's *d*, Pearson's *r*), indicating how they were calculated

Our web collection on statistics for biologists contains articles on many of the points above.

Software and code

Policy information about availability of computer code

Data collection	Molecular modeling was performed with DOCK3.7.1, MODELLER v10.2. CXCalc (ChemAxon's Marvin package Marvin 18.10.0) was used to calculate predominant protomers at relevant pH levels (6.9, 7.4, 7.9). Conformational ensembles were generated with OMEGA (OpenEye, version 2020.2). Machine learning was performed using Python's CatBoost 0.26 package, PyTorch 1.7.1 package and the RangerLars optimizer. The RoBERTa classifier was implemented from the simpletransformers 0.61.6 package. Skorch 0.10.0 package. ZINC15 and Enamine REAL databases are available at: https://zinc15.docking.org and https://enamine.net/compound-collections/real-compounds/real-database. Software used in data generation and analysis can be found at https://github.com/carlssonlab/conformalpredictor.
Data analysis	Python libraries, RDKit 2019_Q3 and OpenEye Toolkits 2020.0.4.

For manuscripts utilizing custom algorithms or software that are central to the research but not yet described in published literature, software must be made available to editors and reviewers. We strongly encourage code deposition in a community repository (e.g. GitHub). See the Nature Portfolio guidelines for submitting code & software for further information.

Data

Policy information about availability of data

All manuscripts must include a data availability statement. This statement should provide the following information, where applicable:

- Accession codes, unique identifiers, or web links for publicly available datasets
- A description of any restrictions on data availability
- For clinical datasets or third party data, please ensure that the statement adheres to our policy

Data are made freely available on Zenodo (10.5281/zenodo.7903160)

Research involving human participants, their data, or biological material

Policy information about studies with human participants or human data. See also policy information about sex, gender (identity/presentation), and sexual orientation and race, ethnicity and racism.

Reporting on sex and gender	N/A
Reporting on race, ethnicity, or other socially relevant groupings	N/A
Population characteristics	N/A
Recruitment	N/A
Ethics oversight	N/A

Note that full information on the approval of the study protocol must also be provided in the manuscript.

Field-specific reporting

Please select the one below that is the best fit for your research. If you are not sure, read the appropriate sections before making your selection.

☒ Life sciences ☐ Behavioural & social sciences ☐ Ecological, evolutionary & environmental sciences

For a reference copy of the document with all sections, see nature.com/documents/nr-reporting-summary-flat.pdf

Life sciences study design

All studies must disclose on these points even when the disclosure is negative.

Sample size	As part of the hyperparameter exploration, the number of samples in the training sets varied from 25,000 to 1,000,000. All other analyses had sample sizes sufficiently large to reproduce computational/experimental values and report error estimates.
Data exclusions	Molecules for which no descriptor could be generated were excluded from the analyses.
Replication	Binding assays (Ki) were done in three independent replicates. Single-concentration radioligand displacements were done in two technical replicates. Computational experiments were done in three replicates where appropriate. All attempts at replication were successful.
Randomization	Random samples were extracted from chemical libraries that were shuffled using Terashuf. Class imbalance was maintained using scikit-learn's stratified split function.
Blinding	Blinding was not applicable to calculations in the study. Standardized experimental procedures did not necessitate blinding.

Reporting for specific materials, systems and methods

We require information from authors about some types of materials, experimental systems and methods used in many studies. Here, indicate whether each material, system or method listed is relevant to your study. If you are not sure if a list item applies to your research, read the appropriate section before selecting a response.

Materials & experimental systems

n/a	Involved in the study
☒	☐ Antibodies
☒	☐ Eukaryotic cell lines
☒	☐ Palaeontology and archaeology
☒	☐ Animals and other organisms
☒	☐ Clinical data
☒	☐ Dual use research of concern
☒	☐ Plants

Methods

n/a	Involved in the study
☒	☐ ChIP-seq
☒	☐ Flow cytometry
☒	☐ MRI-based neuroimaging

Plants

Seed stocks	N/A
Novel plant genotypes	N/A
Authentication	N/A
